# Breaking rules: the complex relationship between DNA methylation and X-chromosome inactivation in the human placenta

**DOI:** 10.1186/s13293-025-00696-6

**Published:** 2025-03-04

**Authors:** Amy M Inkster, Allison M Matthews, Tanya N Phung, Seema B Plaisier, Melissa A Wilson, Carolyn J Brown, Wendy P Robinson

**Affiliations:** 1 BC Children’s Hospital Research Institute, 950 W 28th Ave, Vancouver, BC V6H 3N1 Canada; 2 Department of Medical Genetics, University of British Columbia, 4500 Oak St, Vancouver, BC V6H 3N1 Canada; 3 Department of Pathology & Laboratory Medicine, University of British Columbia, 221 Wesbrook Mall, Vancouver, BC V6T 1Z7 Canada; 4 Center for Evolution and Medicine, School of Life Sciences, Arizona State University, 427 E Tyler Mall, Tempe, AZ 85281 USA

**Keywords:** DNA methylation, X-chromosome inactivation, Placenta, Prenatal development, Sex differences

## Abstract

**Background:**

The human placenta is distinct from most organs due to its uniquely low-methylated genome. DNA methylation (DNAme) is particularly depleted in the placenta at partially methylated domains and on the inactive X chromosome (Xi) in XX samples. While Xi DNAme is known to be critical for X-chromosome inactivation (XCI) in other tissues, its role in the placenta remains unclear. Understanding X-linked DNAme variation in the placenta may provide insights into XCI and have implications for prenatal development and phenotypic sex differences.

**Methods:**

DNAme data were analyzed from over 350 human placental (chorionic villus) samples, along with samples from cord blood, amnion and chorion placental membranes, and fetal somatic tissues. We characterized X chromosome DNAme variation in the placenta relative to sample variables including cell composition, ancestry, maternal age, placental weight, and fetal birth weight, and compared these patterns to other tissues. We also evaluated the relationship between X-linked DNAme and previously reported XCI gene expression status in placenta.

**Results:**

Our findings confirm that the placenta exhibits significant depletion of DNAme on the Xi compared to other tissues. Additionally, we observe that X chromosome DNAme profiles in the placenta are influenced by cell composition, particularly trophoblast proportion, with minimal DNAme variation across gestation. Notably, low promoter DNAme is observed at most genes on the Xi regardless of XCI status, challenging known associations in somatic tissues between low promoter DNAme and escape from XCI.

**Conclusions:**

This study provides evidence that the human placenta has a distinct Xi DNAme landscape, which may inform our understanding of sex differences during prenatal development. Future research should explore the mechanisms underlying the placenta’s unique X-linked DNAme profile, and the factors involved in placental XCI maintenance.

**Supplementary Information:**

The online version contains supplementary material available at 10.1186/s13293-025-00696-6.

## Highlights

We characterized X chromosome DNAme variation in the human placenta relative to other tissues, sample variables (cell composition, ancestry, maternal age, placental weight, fetal birth weight), and patterns of X-chromosome inactivation (XCI).

We confirm a distinct low-methylated landscape of placental X chromosome DNAme, particularly at promoters in XX (female) samples, implicating the inactive X chromosome (Xi).

We observe only a modest (*R* = 0.3) correlation between placental X-linked DNAme and XCI status, which is weaker than observed in other tissues.

**Supplementary Information:**

The online version contains supplementary material available at 10.1186/s13293-025-00696-6.

## Abstract (pa)

In this study, we examine DNA methylation, a DNA modification that can influences gene expression, in the human placenta. The placenta has less DNA methylation overall than most other organs, and here we evaluate DNA methylation patterns on the X chromosome in over 350 placental samples compared to samples of other tissues. Our findings reveal that the placenta has unique DNA methylation patterns on the inactive X chromosome (the second X chromosome in females, which is typically silenced) that differ from what we see in other tissues. We found that DNA methylation on the X chromosome is affected by the cell composition of placental samples, especially trophoblast cells, which are the first cells that contribute to the developing placenta. Interestingly, we observed that genes on the X chromosome in placenta sometimes have low gene expression without high levels of DNA methylation, which suggests that the rules of X chromosome inactivation may differ in the placenta as compared to other tissues. Our research provides insight into how X chromosome DNA methylation in the placenta may influence early development and sex differences before birth. Future studies will need to investigate how these patterns of DNA methylation arise, and what impact they have on prenatal development.

## Background

The placenta is one of the most distinct human organs in terms of its global DNA methylation (DNAme) profile, exhibiting approximately 20% fewer methylated cytosines across the genome compared to other fully differentiated tissues [1]. This DNAme depletion is not uniform across the placental genome, but is concentrated in large regions of low DNAme called partially methylated domains (PMDs) in both sexes [2]. Additionally, in XX (female) placentas, DNAme is specifically depleted at promoters on the inactive X chromosome (Xi) [3]. Understanding the nature of the unique X chromosome DNAme profile in the placenta has the potential to yield insights into the process of X-chromosome inactivation (XCI), and further, may be relevant to our understanding of phenotypic sex differences observed in the prenatal period.

XCI is the process by which one X chromosome in XX cells is epigenetically silenced; in somatic tissues most genes are “subject” to silencing by XCI, while a small percentage “escape” or “variably escape” XCI, and are expressed from both alleles. XCI has been well-studied in somatic tissues, where it is observed that DNAme generally occurs at gene promoters on the inactive X chromosome, and is associated with transcriptional repression of the associated genes [4]. For example, in various tissues (blood, kidney, muscle, and neural tissue), Cotton et al. (2011) found that genes subject to XCI have higher levels of average promoter DNAme in XX samples (β > 0.3) than genes that escape XCI (β < 0.15) [5]. The relationship between DNAme and gene expression from the Xi is robust enough in somatic tissues that promoter DNAme alone predicts XCI status at genes subject to XCI with over 80% accuracy, and at genes escaping XCI with over 70% accuracy [5].

DNAme does not only occur at promoter regions, and it exhibits considerable variability across the entire active and inactive X chromosome in both sexes [6]. Although DNAme plays a crucial role in the genome, the factors driving variation in X chromosome DNAme outside of XCI-related contexts remain poorly understood in most tissues, including the placenta. Studying X chromosome DNAme in the placenta is of particular interest given that the placental villi are largely composed of trophoblast cells derived from the outer trophectoderm layer of the blastocyst. The extraembryonic mesoderm, which derives from hypoblast and/or epiblast after implantation, will contribute to villous mesenchyme, amniotic ectoderm, chorionic mesoderm [7–9]. A small number of cells of the epiblast contribute to the fetus proper, from which adult somatic tissues are derived. Importantly, both the post-implantation wave of epigenetic reprogramming and the process of XCI are thought to occur in humans around the time of implantation, with evidence supporting the fact that XCI likely occurs independently in the different preimplantation cell lineages [7]. As a consequence of the unique developmental origins of the placenta, XCI patterns and X-linked DNAme profiles in the placenta may vary from the patterns commonly observed in somatic tissues.

In this study, we aimed to evaluate patterns of X chromosome DNAme variation in the human placenta and to characterize this variation relative to other tissues, gestational age, and various sample characteristics (biological and technical). We also assessed the extent to which X chromosome promoter DNAme corresponds with previously-reported XCI status in the placenta, based on allele-specific gene expression evidence reported by Phung et al. (2022) [10]. By comparing patterns of X-linked DNAme in the placenta to somatic tissues originating from the inner cell mass, our findings provide valuable insights into the regulatory mechanisms important to XCI in different tissue contexts, and have the potential to provide broader insights into how phenotypic sex differences may manifest during prenatal development.

## Results

### Cohort construction

DNA methylation (DNAme) array data from several datasets were arranged into three cohorts: (i) a *multi-tissue* dataset (Table 1) comprised of placental samples from all trimesters of pregnancy, feto-placental membranes from the third trimester (chorionic membrane, amniotic membrane), second trimester fetal somatic tissues (brain, kidney, muscle, spinal cord), and term cord blood samples - all profiled with either the Illumina Infinium MethylationEPIC v1.0 (EPIC) or Illumina Infinium HumanMethylation450 (450K) arrays; (ii) a *discovery cohort* of normative placentas across gestation, profiled with the EPIC array (Table 2); and (iii) a *replication cohort* of normative term placentas, profiled with the 450K array (Supplementary Table 1). Full cohort construction and data processing steps are detailed in the Methods.

**Table 1:** Counts of samples included in multi-tissue dataset from Illumina Infinium HumanMethylation450 (450K) and Infinium Methylation EPIC (EPIC) array datasets. GEO dataset owner refers to the contributing author of the Gene Expression Omnibus (GEO) dataset

	XX (female)	XY (male)	Datasets	GEO Dataset Contributor
Samples with 450K data				
**Brain**	5	13	GSE69502	Robinson
**Spinal cord**	12	18	GSE69502	Robinson
**Kidney**	17	17	GSE69502	Robinson
**Muscle**	15	14	GSE69502	Robinson
**Placenta (Chorionic villi)**				
2nd trimester	18	15	GSE69502	Robinson
Term	12 (RICHS) 12 (NHBC) 12 (EPIC)	12 (RICHS) 12 (NHBC) 12	GSE71678 (RICHS), GSE75248 (NHBC), GSE55196, GSE98224, GSE100197, GSE108567, GSE281173	Marsit Marsit Robinson Robinson Robinson Robinson Robinson
**Cord blood (term)**	36	36	GSE151042	Plusquin
**Sorted cord blood** (CD4 and CD8 T cells, granulocytes, monocytes, and natural killer cells)	28	12	GSE68456	Robinson
Samples with EPIC data				
**Amnion**	5	7	GSE115508	Robinson
**Chorion**	6	7	GSE115508	Robinson
**Placenta (Chorionic villi)**				
1st trimester	3	5	GSE281173	Robinson
2nd trimester	9	6	GSE281173	Robinson
3rd trimester	19	16	GSE115508	Robinson
Term	93	109	GSE232778	Robinson
**Cord blood (term)**	12	10	GSE224339	Fradin

**Table 2:** Sample characteristics of the discovery cohort, profiled with the Illumina Infinium MethylationEPIC array

	XX (female)	XY (male)	*p* value^*^
**Sample size (n)**	107	121	
**Collection location**			
Vancouver, CA (n (%))	61 (57.0)	62 (51.2)	ns
Queensland, AU (n (%))	46 (43.0)	59 (48.8)	
**Cohort**			
V-NORM	30 (28.0)	29 (24.0)	ns
V-SSRI	31 (29.0)	33 (27.3)	
QF2011	46 (43.0)	59 (48.8)	
**Maternal age (mean (SD))**	32.3 (5.4)	32.6 (5.1)	ns
**Gestational age at delivery (mean (SD))**	36.9 (7.2)	37.2 (7.3)	ns
**Trimester**			
First (1–12 weeks) (n (%))	3 (2.8)	5 (4.1)	ns
Second (13–24 weeks) (n (%))	9 (8.4)	6 (5.0)	
Third (25–36 weeks)	4 (3.7)	5 (4.1)	
Term (37–42 weeks) (n (%))	91 (85.0)	105 (86.8)	
**Birthweight (g**,** mean (SD))**			
First trimester (mean (SD))	-	-	ns
Second trimester (mean (SD))	-	-	
Third trimester (mean (SD))	2864.7 (1127.0)	3104.3 (667.2)	
Term (mean (SD))	3458.9 (480.3)	3598.6 (399.2)	
**Birthweight Z-score** ^**†**^ **(mean (SD))**			
First trimester (mean (SD))	-	-	ns
Second trimester (mean (SD))	-	-	
Third trimester (mean (SD))	0.39 (0.43)	-0.10 (1.28)	
Term (mean (SD))	0.15 (0.91)	0.13 (0.82)	
**Placental weight in grams (mean (SD))**			
Second trimester (mean (SD))	114.0 (0.0)	116.4 (33.5)	ns
Third trimester (mean (SD))	691.5 (56.1)	635.2 (127.8)	
Term (mean (SD))	616.0 (132.0)	639.8 (114.1)	
**PlaNET Ancestry** ^**^			
Coordinate 1 (mean (SD))	0.03 (0.1)	0.03 (0.1)	ns
Coordinate 2 (mean (SD))	0.15 (0.3)	0.14 (0.3)	
Coordinate 3 (mean (SD))	0.82 (0.3)	0.83 (0.3)	
**Estimated cell type proportions** ^**††**^			
nRBC (mean (SD))	0.00 (0.01)	0.00 (0.01)	ns
Hofbauer (mean (SD))	0.02 (0.01)	0.02 (0.010	ns
Endothelial (mean (SD))	0.08 (0.02)	0.08 (0.03)	ns
Stromal (mean (SD))	0.11 (0.09)	0.11 (0.08)	ns
Cytotrophoblast (mean (SD))	0.19 (0.06)	0.17 (0.06)	< 0.05
Syncytiotrophoblast (mean (SD))	0.59 (0.10)	0.61 (0.11)	ns

^*^p values represent significance of ANOVAs for continuous variables and Chi square tests for categorical variables

^†^Birthweight Z-score refers to the number of standard deviations away from the sex- and gestational age-specific Canadian population mean presented in Kramer et al. (2001) [11]

^**^Genetic ancestry was estimated using the PlaNET R package, and is described on three compositional axes, Coordinate 1 reflects probability of African ancestry, Coordinate 2 reflects probability of East Asian Ancestry, and Coordinate 3 reflects probability of European ancestry

^††^Cell composition of bulk chorionic villus tissue estimated using the PlaNET R package

### The human placenta has a distinct X chromosome DNA methylation profile

Our first aim was to understand global patterns of DNAme variability between tissues. Accordingly, we used the multi-tissue cohort (Table 1) to conduct principal components analysis (PCA) on autosomal and X-linked DNAme. For these analyses, PCA was conducted separately in XX and XY samples (sex-stratified).

**Fig. 1:**
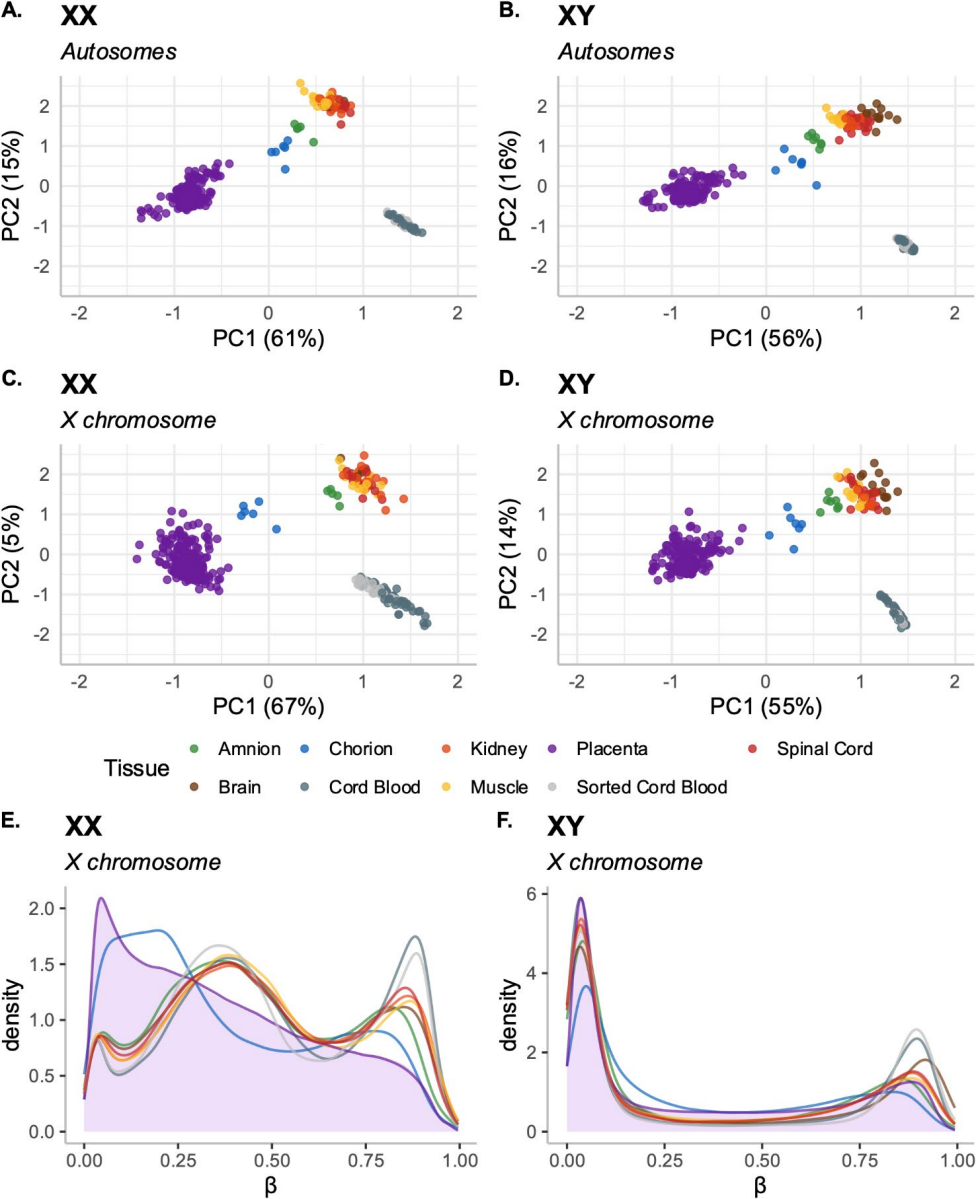
Autosomal and X chromosome DNAme characteristics of XX samples. For plots **A**-**D** each point is a sample plotted along Principal Component 1 (PC1, x axis) and PC2 (y axis), points are colored by tissue of origin. Each plot shows standardized PC scores (mean-centred and scaled by the standard deviation), to allow comparison across datasets. Identical axis limits are utilized for consistent scaling between plots, and the percentage of variance explained by each PC is indicated in the axis label. **(A)** Principal components analysis (PCA) of all autosomal probe-filtered CpGs in XX samples from the multi-tissue dataset. **(B)** Principal components analysis (PCA) of all autosomal probe-filtered CpGs in XY samples from the multi-tissue dataset. **(C)** Principal components analysis (PCA) of all probe-filtered X chromosome CpGs in XX samples from multi-tissue datasets. **(D)** Principal components analysis (PCA) of all probe-filtered X chromosome CpGs in XY samples from the multi-tissue dataset. **(E)** DNAme density distribution of probe-filtered X chromosome CpGs in XX samples from the multi-tissue dataset, each line represents the average density of samples occupying a particular beta value in each tissue, lines are colored by tissue of origin and the density distribution for placenta is filled in (purple). **(F)** DNAme density distribution of X chromosome in XY samples, lines are colored by tissue of origin and the density distribution for placenta is filled in (purple)

In both XX and XY samples, the first two principal components (PC1 and PC2) of the autosomal and X chromosome DNAme explained > 70% of the observed variance, and were significantly associated with tissue, Fig. 1A-D (p value < 0.05 for linear models of PC1 ~ Tissue and PC2 ~ Tissue). In both sexes, PC1 separated placental samples from somatic tissues, while PC2 separated blood from other somatic tissues, suggesting that tissue has a similar impact on autosomal and X-linked DNAme, and a similar effect in both sexes. While it is known that autosomal DNAme varies by tissue [12–15], the observation of X chromosome DNAme variation by tissue (particularly placenta versus somatic) is relatively novel.

In all PC1 versus PC2 scatterplots, samples from the amnion and chorion were positioned between placenta and fetal somatic tissues, with chorion samples falling closer to placenta, and amnion samples closer to fetal somatic tissues, Fig. 1A-D. The positioning of amnion and chorion samples in the PC scatterplots is consistent with their developmental origins, as some layers of each amnion and chorion share a common cellular origin (extraembryonic mesoderm), while the other layer(s) of chorion derive from trophectoderm, and the other amnion layers derive from embryonic ectoderm [8]. Additionally, the separation of blood samples from other somatic tissues in Fig. 1A-D has been previously observed in clustering analyses of prenatal tissues [16], although the factors driving this blood/somatic tissue difference are unclear and may be related to the early origins of blood from yolk sac.

As many datasets contributed to this analysis, we next sought to evaluate the relative extent to which dataset versus tissue effects contributed to sample separation in PCA. We applied the principal component partial R-square (PC-PR2) method to the X chromosome DNAme data in XX samples [17, 18], collapsing both dataset and tissue to simplified representative variables: dataset was collapsed to reflect GEO dataset contributor (see Table 1: Fradin, Marsit, Plusquin, Robinson), while tissue was collapsed into (i) placenta, (ii) fetal membranes (amnion, chorion), (iii) cord blood (cord blood, sorted cord blood), and (iv) fetal somatic tissues (brain, kidney, muscle, spinal cord). By PC-PR2, tissue contributed to 38.5% and dataset to 27.9% of X chromosome DNAme variance in XX samples assessed (*n* = 317), confirming that tissue effects primarily drive the sample separation observed in X chromosomal PCA, Supplementary Fig. 1A.

We subsequently examined the DNAme β value density distributions on the autosomes and X chromosome across tissues. In both sexes, autosomal DNAme β value distributions largely overlapped among tissues, including placenta (Supplementary Fig. 1B and C). In contrast, the X chromosome DNAme distribution did not overlap between placenta and other tissues, particularly in XX samples, Fig. 1E. In most tissues, X chromosome DNAme β values were distributed trimodally, representing a mixture of CpGs with DNAme at 0, 1, or 2 alleles (β values of roughly 0, 0.5, and 1). In XX samples, the distribution of X chromosome β values in placenta was left-shifted toward more low and intermediate DNAme, while XX chorion samples showed a similar but less-pronounced left shift towards lower/intermediate DNAme, Fig. 1E. In XY samples, the X chromosome DNAme density distributions were more consistent across tissues, with no separation of placenta or chorion from the other tissue distributions, Fig. 1F. These plots therefore emphasize the distinct profile of X-linked DNAme in XX placentas and trophoblast cells in particular.

All PCA analyses described to this point were conducted in sex-stratified subsets of the multi-tissue dataset. When PCA was run in the full (mixed-sex) multi-tissue dataset, autosomal DNAme separated samples primarily by tissue along PC1 and PC2, similar to what observed in sex-stratified PCA (Supplementary Fig. 1D). However, on the X chromosome, mixed-sex PCA plots showed first a separation by sex along PC1, and subsequently a separation by tissue along PC2 (Supplementary Fig. 1E). This is expected, given that the X chromosome DNAme signature in XX samples arises from the combined signatures of the active and inactive X chromosomes (Xa and Xi), relative to the single X in XY samples (Xa). The Xa and Xi are known to have distinct DNAme profiles, leading to overall sex-differential patterns of DNAme measured by arrays [3, 6]. However, these results underscore our observation that on both the autosomes and X chromosome, in both sexes, DNAme variation is strongly associated with tissue.

### The human placenta has less Xi promoter DNAme than somatic tissues

Cotton et al. (2009) used the Illumina GoldenGate array to identify that the X chromosome in placenta had lower promoter DNAme than the X in adult blood, particularly in XX samples, which implied lower promoter DNAme on the Xi relative to the Xa [3]. The newer 450K and EPIC arrays profile tens of thousands of X-linked CpGs, compared to the 84 profiled by GoldenGate, enabling a more comprehensive characterization of X chromosome DNAme in placenta. For this analysis, we compared promoter DNAme in term placental samples to somatic tissues (cord blood, placental amniotic membrane (amnion), and fetal muscle). Placental chorionic membrane samples were excluded from this analysis due to their trophoblast-derived component.

**Fig. 2:**
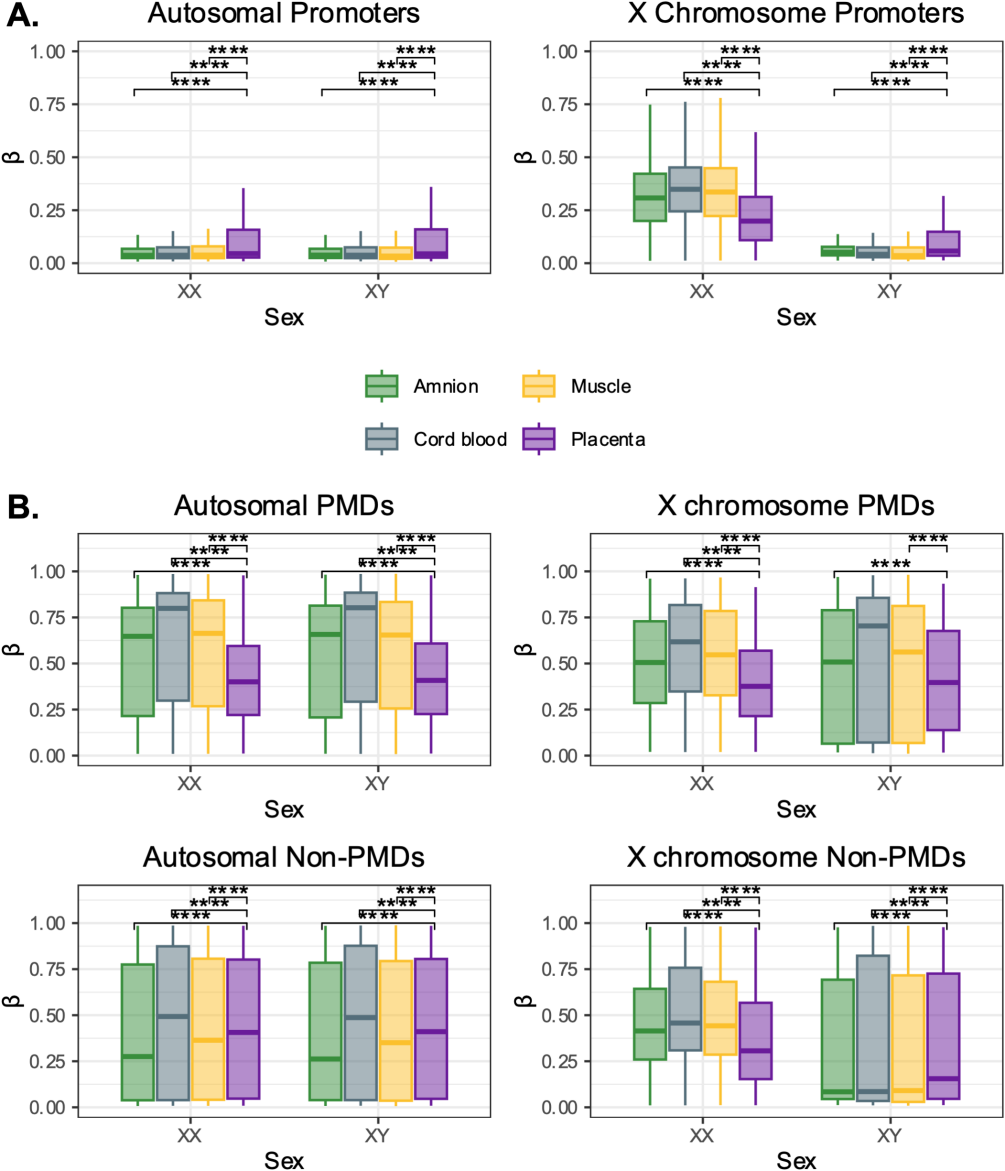
DNAme at promoters and partially-methylated domains in term placenta as compared to amnion and somatic tissues. For all plots, boxplots display mean DNAme in XX and XY samples, with boxes drawn between the 25th -75th centiles of the data and horizontal lines at the mean, whiskers indicate 1.5x the interquartile range past the 25th and 75th centiles. Wilcoxon tests were performed between placenta and all other tissues, adjusted p values are indicated where mean DNAme differs by tissue. **** indicates FDR < 0.0001, *** FDR < 0.001, ** FDR < 0.01, * FDR < 0.05, non-significant comparisons are not shown. **(A)** Boxplots of mean promoter DNAme at autosomal (left) and X chromosome (right) loci. **(B)** Boxplots of mean DNAme in partially-methylated domains (PMD, top row) and non-partially-methylated domain regions (non-PMD, bottom row), on the autosomes (left) and X chromosome (right)

Our analysis revealed that promoter DNA methylation (DNAme) profiles were similar across tissues on the autosomes (both sexes) and the X chromosome in XY samples. While placenta exhibited slightly higher average promoter DNAme on the autosomes than other tissues in both sexes, these differences were minimal (Fig. 2A). The X chromosome in XX samples consistently showed higher average promoter DNAme relative to the autosomes (in both sexes) or to the X in XY samples, reflecting the contribution of DNAme of Xi promoters in XX samples (Fig. 2A). However, comparing XX samples across tissues, placenta had lower X-linked promoter DNAme than any other tissue (XX β_mean chrX_ placenta = 0.23, amnion = 0.33, cord blood = 0.35, muscle = 0.33; Wilcoxon tests of placenta versus all other tissues FDR < 0.0001), consistent with Cotton et al.’s observation of Xi DNAme depletion in placenta.

These findings confirm lower Xi promoter DNAme in placenta relative to other tissues, and indicate that the X in XY samples and the autosomes in both sexes have comparable DNAme levels between placenta and other tissues. The consistency between autosomal DNAme and the X in XY samples, which is often used as a proxy for the Xa [4], further confirms that placental promoter DNAme primarily differs from other tissues on the Xi.

Low DNAme at autosomal loci in the placenta has been largely attributed to the presence of partially-methylated domains (PMDs), or large blocks of low DNAme relative to the surrounding genomic context. PMDs are uniquely observed in placenta, some cultured cell types, and cancer [1, 2, 16]. PMDs have been characterized across the placental genome, including on the X chromosome [2], though the extent to which PMDs explain Xi DNAme depletion in placenta relative to other tissues has not been explicitly tested.

We used PMD coordinates defined in [2], to first confirm lower PMD DNAme in placenta compared to other tissues, which we observed in both sexes on the autosomes and on the X chromosome (Fig. 2B). Outside of PMDs, only XX samples had consistently lower X chromosome DNAme in placenta relative to other tissues. These results indicate that while PMDs explain a large portion of low DNAme observed in the placenta, the Xi DNAme profile observed in placenta is not completely explained by PMD occupancy.

LINE-1 repetitive elements have also been noted to have low DNAme in the placenta, though it has since been reported that PMD occupancy of LINE-1 elements drives this finding on the autosomes [2, 19]. As both LINE-1 and SINE elements have been postulated to be relevant in XCI spreading [20], we therefore assessed DNAme at both LINE-1 and SINE elements on the X chromosome in placenta relative to other tissues, and relative to PMD occupancy.

First, we confirmed that low DNAme at LINE-1 elements in placenta was largely explained by PMD overlap, with similar pattern observed in SINEs, namely that outside of PMDs the difference between placenta and somatic DNAme is small (Supplementary Fig. 2A-D). On the X chromosome, however, placenta had lower DNAme than somatic tissues both in PMDs and outside of PMDs. To analyze the combined effects of PMDs and repetitive elements on X chromosome DNAme, we subsequently grouped CpGs into “repetitive” (LINE or SINE) and “non-repetitive” regions, as well as “PMD” and “non-PMD” regions (Supplementary Fig. 3). When focusing specifically on CpGs in non-repetitive and non-PMD regions, we observed that DNAme was substantially lower in placenta than in other tissues, and this effect was most pronounced on the X chromosome in XX samples (XX β_mean chrX_ placenta = 0.34, somatic = 0.43; XX β_mean autosomal_ placenta = 0.42, somatic = 0.44; Wilcoxon tests of placenta versus all other tissues FDR < 0.0001). These results therefore suggest that low Xi DNAme in placenta is not fully explained by patterns associated with either PMD or LINE-1/SINE repetitive element occupancy.

To further investigate the pattern of low DNAme on the placental X chromosome relative to other tissues, we used the CentriMo tool from MEME Suite [21–23] to test for enrichment of transcription factor binding motifs in regions of low placental DNAme. Specifically, 100 base-pair windows around the 1,364 CpGs on the X chromosome with average XX DNAme ∆β > 0.20 (∆β = somatic – placenta) were subjected to CentriMo analysis relative to the background of all processed X chromosome CpGs (*n* = 8,676), using the Homo sapiens Comprehensive Model Collection (HOCOMOCO) version 11 transcription factor binding motif database. No transcription factor binding motifs were differentially enriched between the two sets of sequences, indicating that regions with low X chromosome DNAme in placenta relative to other tissues reflect a similar distribution of transcription factor binding motifs found across the broader X chromosome (*n* = 18, Supplementary Table 3).

### Placental Xi DNAme is stable across gestation

**Fig. 3:**
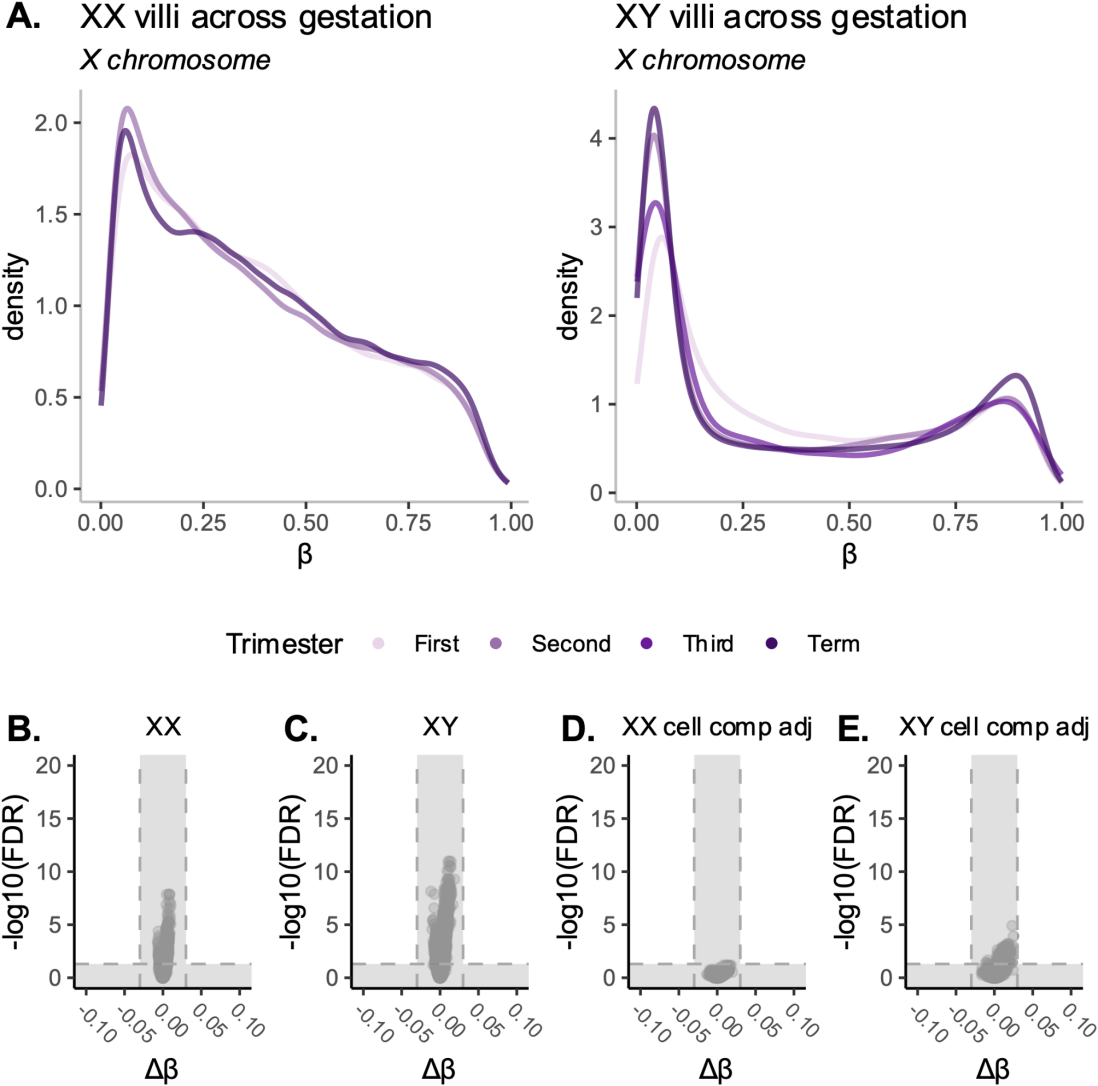
Placental X chromosome DNAme profiles across gestation. **(A)** Density plots of DNAme on the X chromosome from first, second, third trimester, and term samples, in XX (left) and XY (right) placenta. **(B)** Volcano plot of linear model in XX samples, evaluating DNAme at X chromosome CpGs associated with gestational age. Each point on the volcano plot is a single CpG, difference in DNAme with one week change in gestational age is plotted along the X axis (∆β) and the -log10 of the FDR is plotted along the Y axis. Non-significant areas are shaded in grey (under FDR < 0.05, and absolute ∆β < 0.03). **(C)** Volcano plot in XY samples evaluating X-linked DNAme associated with gestational age. **(D)** Volcano plot in XX samples evaluating X-linked DNAme associated with gestational age, adjusted for cell composition. **(E)** Volcano plot in XY samples evaluating X-linked DNAme associated with gestational age, adjusted for cell composition

To evaluate whether the low levels of Xi DNAme observed in placenta were consistent or acquired across the course of gestation, we analyzed placental samples from all trimesters of pregnancy (Table 2; Fig. 3A and B), and ran sex-stratified linear models to identify X-linked DNAme associated with gestational age (continuous) in both XX (*n* = 114) and XY (*n* = 122) samples. At the 16,162 X chromosome CpGs analyzed, we found no significant associations with gestational age in either sex (Fig. 3B and C). This lack of association persisted after adjustment for cell composition, confirming that known changes in placental cell composition across gestation (Fig. 3D and E) [24, 25] were not masking our ability to detect X chromosome DNAme alterations throughout pregnancy. Taken together, our findings indicate the stability of X chromosome DNAme profiles in the placenta across gestation.

### Cell composition is associated with variability in placental Xi chromosome DNAme

An extensive body of research has investigated autosomal DNAme variation in the placenta associated with pregnancy complications and maternal/fetal exposures [12, 26–38], and phenotypes including biological sex [39, 40], birth weight [41] and gestational age at birth [42, 43]. However, previous analyses have been restricted to the autosomes, and relatively little is known about how technical and biological factors affect placental X chromosome DNAme variation. To address this question, we sought to assess how high-level patterns of X chromosome DNAme variability (PCA) in the placenta were associated with biological and technical sample characteristics including birth weight, placental weight, and maternal age; these analyses were restricted to term XX placental samples from the discovery cohort (*n* = 93) as these samples had the most available extended sample information. All other tissues were excluded from this analysis.

**Fig. 4:**
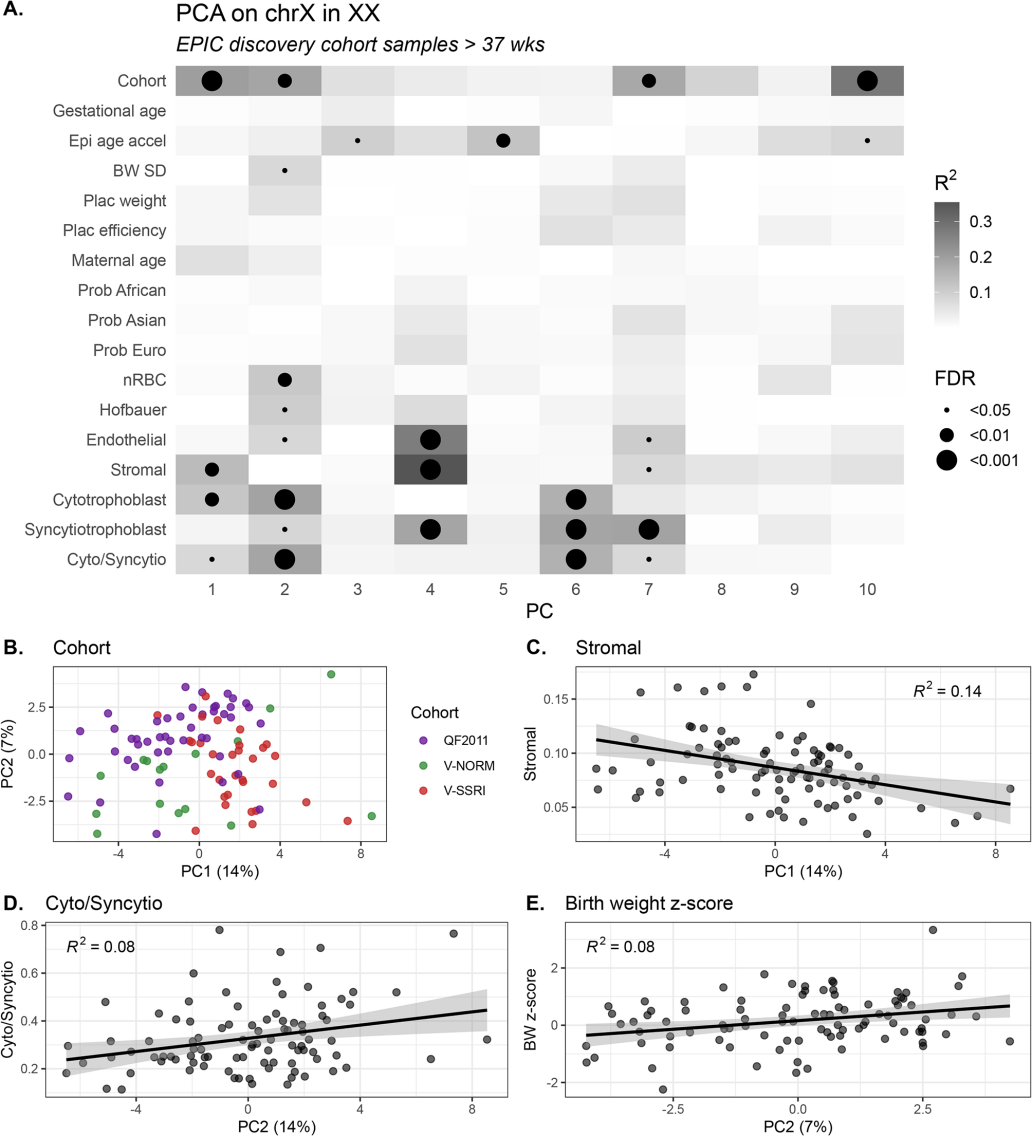
Association of sample characteristics with X chromosome DNAme principal components, in XX placental samples at term (*n* = 93). (**A**) Heatmap of the strength of association (PC ~ variable), shading reflects R^2^ while black dots represent significant associations after multiple test correction (FDR < 0.05). Epi age accel refers to epigenetic age acceleration, Plac refers to placenta, BW SD refers to the z-score of birth weight for sex and gestational age, Prob African/Asian/European refer to the three continuous ancestry components estimated by PlaNET, and Cyto/Synctio reflects the ratio of estimated cytotrophoblast over syncytiotrophoblast cells, per sample. (**B**) Scatterplot of PC1 versus PC2 for all samples, colored by sub-cohort membership. (**C**) Scatterplot of PC1 versus stromal cells. (**D**) Scatterplot of PC1 versus cytotrophoblast to syncytiotrophoblast ratio. (**E**) Scatterplot of PC2 versus birth weight standard deviation z-score

On the X in XX placentas, the first four PCs accounted for 31% of X chromosome DNAme variation (PC1 14.0%, PC2 7.2%, PC3 6.5%, PC4 3.5%), the first ten PCs accounted for 42% of the variance. To assess the association between DNAme variation (PCs) and sample characteristics, linear regression models were fit for each PC + demographic variable pair, of the form PC ~ variable, Fig. 4A. Notably, PC1 (14%) was associated with cohort (R^2^ = 0.2, FDR < 0.001), Fig. 4B, reflecting the three sub-cohorts comprising the discovery cohort (V-NORM, V-SSRI, QF2011, see Methods). PC1 was also associated cell type proportions, including stromal and cytotrophoblast cell proportions (R^2^ = 0.14 and R^2^ = 0.11, respectively, both FDR < 0.01), and the cytotrophoblast-to-syncytiotrophoblast ratio (R^2^ = 0.08, FDR < 0.05), Fig. 4C and D. PC2 (7.2%) was associated with cohort (R^2^ = 0.19, FDR < 0.01), birth weight z-score (R^2^ = 0.08, FDR < 0.05), Fig. 4E, and cell composition (FDR < 0.05 for all cell types other than stromal (n.s.), notably cytotrophoblast R^2^ = 0.19, FDR < 0.001). PC3 (6.5%) was associated with epigenetic age acceleration, i.e. the residual of epigenetic age regressed on chronological gestational age (R^2^ = 0.10, FDR < 0.05), and PC4 (3.5%) was associated with the proportions of endothelial, stromal, and syncytiotrophoblast cells (*R* = 0.27, 0.35, and 0.19, respectively, all FDR < 0.001).

In the full cohort, the first two PCs were associated with cell composition and birth weight z-score, variables that themselves differ slightly between the three sub-cohorts. For context, the birth weight z-score measures how many standard deviations offspring birth weight deviates from the mean birth weight of peers matched for sex and gestational age, based on the Canadian standard reference [11]. To understand the cohort-confounded associations observed in PCA, we therefore investigated X chromosome DNAme by PCA within each sub-cohort (V-NORM, V-SSRI, QF2011). We found that in each of the three sub-cohorts, a subset of cell composition metrics were nominally associated with the top few PCs (Supplementary Fig. 4A-D), notably with endothelial and cytotrophoblast cells in QF2011, Hofbauer and stromal cells in V-NORM, and Hofbauer, cytotrophoblast, and syncytiotrophoblast cells in V-SSRI. The significance of association between any of the first four PCs and birthweight z-score was attenuated in the sub-cohort PCA studies, though this metrics may be interesting to assess in larger cohorts. The relative consistency in variables associated with the top PCs in each sub-cohort thus provided good evidence that cohort-level variability was not driving the cell-associated variation patterns observed in the larger combined dataset.

### Placental X chromosome DNAme is moderately correlated with silencing

When studying X chromosome DNAme variation, it is critical to consider the relationship between DNAme and XCI. In somatic tissues, genes subject to XCI consistently have promoter DNAme in XX samples (β values > 0.3), while genes that escape XCI largely have low promoter DNAme (β values < 0.2) [4]. We therefore hypothesized that a similar relationship between increased promoter DNAme and an increased likelihood of a gene being subject to XCI would exist in the placenta, and sought to evaluate this relationship directly by cross-referencing to an external study that established patterns of placental XCI using allele-specific expression (ASE) in term chorionic villi [10].

In the Phung et al. (2022) gene expression study [10], heterozygous variants on the X chromosome in term XX placentas were used to calculate median allele balance per gene as an indication of the level of escape from XCI, as genes that escape XCI are expressed from both alleles (expected allele balance ~ 0.5). In the original study, a gene was considered “subject” to XCI if highly skewed expression of one allele was observed in a majority of XX samples (allele balance of ≥ 0.8 in > 70% of samples, and median allele balance of ≥ 0.8 across all samples). A gene was called “escape” from XCI if highly skewed expression was observed in a minority of samples (< 30% of samples with allele balance ≥ 0.8, and median allele balance across all samples ≤ 0.75). Genes falling between these extremes were deemed “variable escape”. Per gene, XCI status was summarized as a single continuous value, reported as the proportion of informative samples in which a gene was measured to be subject to XCI, a metric which we hereafter refer to as “proportion subject”.

For all genes on the X chromosome covered in both [10] and our DNAme data (term discovery cohort samples), we assessed the correlation between mean/median DNAme per gene and the proportion of samples in [10] in which the same gene was subject to XCI (proportion subject). Overall, DNAme data was available for the promoters of 136 genes covered in [10] (*n* = 622 CpGs), and gene body DNAme was available for 174 genes (*n* = 3,639 CpGs).

First, we found that mean promoter DNAme was weakly correlated with proportion subject (*R* = 0.18, FDR = 0.06). At all genes examined, mean gene body DNAme was less correlated with proportion subject than was promoter DNAme (DNAme *R* = -0.05, FDR = 0.60).

We then focused on factors that could affect the strength of the promoter DNAme relationships. We observed similar correlations between proportion subject and both mean and median promoter DNAme (mean: *R* = 0.18, FDR = 0.06, median: *R* = 0.16, FDR = 0.08). The mean sex difference in promoter DNAme (∆β = XX - XY) was more strongly correlated with proportion subject (*R* = 0.27, FDR < 0.05) than was mean promoter XX DNAme alone. The correlation further improved after excluding genes with high promoter DNAme in XY samples (β > 0.1, *n* = 27/136, 20%) (*R* = 0.29, FDR < 0.05), and including only promoters that overlapped high (HC) or intermediate (IC) density CpG islands (*n* = 112/136, 86%) (*R* = 0.33, FDR < 0.05).

**Fig. 5:**
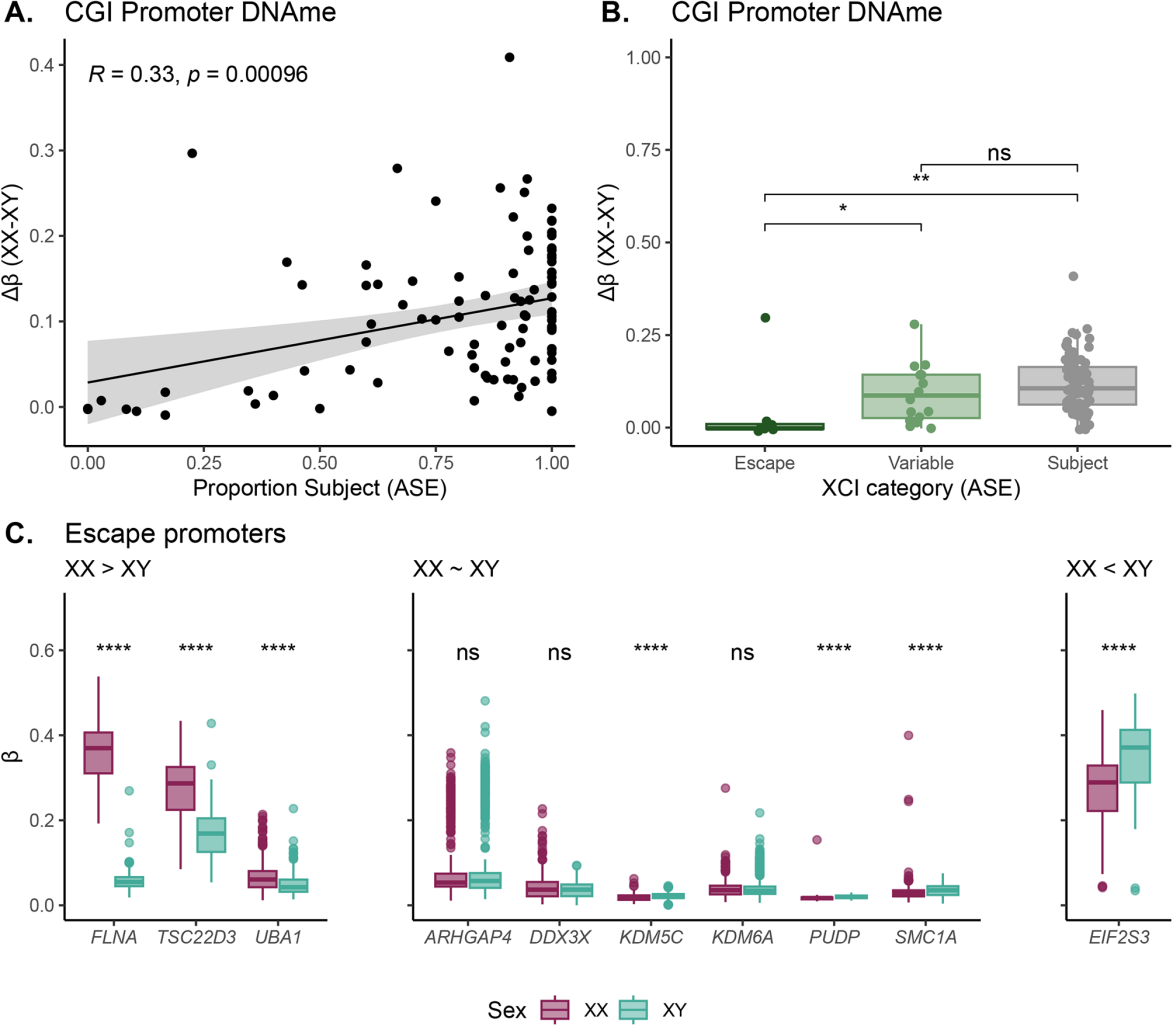
Relationship between DNAme and XCI status from allele-specific expression (ASE) data. **(A)** Correlation between sex difference in DNAme at X-linked promoters and proportion subject, considering only promoters that overlap CpG islands and have XY DNAme β < 0.10. Pearson correlation coefficient and p value are shown in the top left corner, a line of best fit is shown in blue with 95% confidence intervals shaded in grey. **(B)** Mean sex difference in DNAme at X-linked promoters of genes that are categorized as escape, variable escape, or subject to XCI in [10]. Wilcoxon test adjusted p values (FDR) for XX versus XY comparisons are shown at top of plots, ** indicates FDR < 0.01, *** indicates FDR < 0.001, **** indicates FDR < 0.0001, n.s. indicates not significant. **(C)** For all ASE escape genes, boxplots showing mean XX and mean XY promoter DNAme β values, Wilcoxon test adjusted p values (FDR) for XX versus XY comparisons are shown at top of plots, ** indicates FDR < 0.01, *** indicates FDR < 0.001, **** indicates FDR < 0.0001, n.s. indicates not significant

In summary, we observed that the correlation between X chromosome promoter DNAme and proportion subject in placenta was modest (*R* ~ 0.3) (Fig. 5A, Supplementary Table 2). The approximate strength of association between promoter DNAme and proportion subject replicated in the independent 450K term replication cohort (*R* = 0.30, FDR < 0.05) (Supplementary Fig. 5A), indicating between-dataset consistency in the placental DNAme landscape at X-linked promoters. Further, to ensure that variation within the discovery cohort (composed of three sub-cohorts) did not confound associations between X-linked promoter DNAme and proportion subject, we tested and observed consistent DNAme-ASE relationships within each sub-cohort, see Supplementary Fig. 6.

While the “proportion subject” metric reflects the number of individuals in [10] in which a gene was subject to XCI, we also sought to evaluate whether mean/median DNAme in the placenta would more strongly correlate with the median allele balance per gene (of all samples in [10]). The median allele balance metric does not depend on the categorical XCI status determined in the original manuscript, and thus we hypothesized may show a different relationship with promoter DNAme. When the correlations between DNAme values and median allele balance were evaluated, the results were very similar to the DNAme-proportion subject results, see Supplementary Table 2. We additionally analyzed whether promoter DNAme correlated with the total expression of genes on the X chromosome (not allele-resolved) using data from [44] which analyzes the same cohort described in Phung et al. (2022), and found a weakly negative relationship between promoter DNAme and X-linked gene expression (log2CPM). However, a similar promoter DNAme ~ log2CPM relationship was observed in both sexes, see Supplementary Fig. 7. These results therefore indicate that low XX promoter DNAme in placenta is not reflective of total gene expression patterns (not allele-resolved) from the X chromosome.

To this point, our analyses of promoter DNAme relative to XCI status were based on promoters defined using ENCODE’s functional annotation of promoter-like sequences [45]. To determine if the functional annotation of promoters affected the correlation with proportion subject, we examined base pair windows around the transcription start site (TSS), regardless of promoter annotation. We found that the mean sex difference in DNAme in the TSS200-1500 region (*n* = 1,500 CpGs) was similarly correlated with proportion subject (*R* = 0.25, FDR < 0.05) as were annotated promoter DNAme levels. In contrast, DNAme in the TSS200 alone was not correlated with proportion subject (*R* = 0.14, FDR = 0.07). When focusing solely on CpGs in the TSS200-1500 that were not in functionally-annotated promoters (*n* = 855 CpGs), the correlation between DNAme and proportion subject remained significant (*R* = 0.25, FDR < 0.05).

Next, we evaluated DNAme at enhancers, using ENCODE’s annotation for proximal enhancer-like sequences (pELS) [45]. We examined 941 unique pELS regions (*n* = 1,458 CpGs), and observed that mean enhancer DNAme was correlated with proportion subject (*R* = 0.19, FDR < 0.05). The correlation improved when considering the mean sex difference in DNAme (*R* = 0.25, FDR < 0.05), similar to what we observed in promoters. To ensure that the relationship between enhancer DNAme and proportion subject were distinct from promoter DNAme relationships, we considered the overlap between promoter and enhancer regions and found no common CpGs, confirming the independence of enhancer DNAme observations.

Overall, these results suggest that the correlation between DNAme and proportion subject in the placenta is consistent in promoter and TSS regions (*R* ≈ 0.3) regardless of functional promoter annotation, indicating that DNAme variation upstream of TSSs provides some degree of insight into XCI status. Additionally, our results indicate that proportion subject also correlates with enhancer DNAme. For both promoters and enhancers, the strongest associations with XCI were observed when considering the mean sex difference in DNAme, which accounts for DNAme arising from the Xa and isolates a signature more reflective of the Xi.

We then considered whether promoter DNAme could distinguish genes based on their categorical XCI status in placenta, as is possible in somatic tissues [5]. We compared the average sex difference in promoter DNAme (∆β) between genes that escape, variably escape, or are subject to XCI in placenta, as categorized in [10], see Fig. 5B. Escape genes generally showed small sex differences in promoter DNAme, and were significantly different from genes in the subject and variable escape categories (Wilcoxon test FDR < 0.05). However, genes in the subject and variable escape categories were not distinct from each other by mean promoter DNAme (Wilcoxon test FDR > 0.05). Notably, within the escape category, the *FLNA* promoter exhibited a substantial sex difference in DNAme (∆β = 0.30), with mean XX and XY β values of 0.36, and 0.06, respectively (Fig. 5C). Despite *FLNA* showing evidence of XCI escape based on ASE in 31/40 informative samples in [10], its large sex difference in DNAme suggests DNAme accumulation on the Xi in XX samples. Other escape genes, such as *EIF2S3* (mean XX β = 0.27, mean XY β = 0.35), and *TSC22D3* (mean XX β = 0.27, mean XY β = 0.17), had high mean promoter DNAme in both sexes. However, *EIF2S3* had higher XY than XX promoter DNAme, indicating DNAme on the Xa at this gene. The mechanism and impact of XY > XX DNAme are not known, but may be related to lack of erasure of DNAme in the maternal germline, and may have implications for gene expression in XY placentas [46].

### Relationship between sex and X chromosome DNAme in cord blood versus placenta

To compare the relationship between DNAme and XCI status observed in placenta to that observed in blood, we considered 78 promoters covered by DNAme data with XCI statuses reported in both tissues [10, 47]. Categorical XCI statuses (escape, variable escape, and subject) were utilized for these analyses to harmonize XCI metrics between tissues.

At these 78 genes, the number of genes per XCI category was similar between placenta and cord blood (Chi-square p value > 0.05; placenta: n_escape_ = 4, n_variable_ = 7, n_subject_ = 67, cord blood: n_escape_ = 7, n_variable_ = 1, n_subject_ = 70). Additionally, within each XCI category, the majority of genes were categorized similarly in both tissues (n_escape_ = 3, n_subject_ = 64). Only one gene that escaped in placenta was reported to be subject to XCI in blood (*FLNA*), while two blood-escape genes were subject to XCI in placenta (*PRKX*, *VGLL1*). The remaining eight genes were reported to variably escape in one tissue and were classified as either escape or subject to XCI in the other tissue (*ATRX*, *CHRDL1*, *EGFL6*, *MAGEA8*, *MBTPS2*, *SMS*, *TXLNG*, *ZXRX2*).

**Fig. 6:**
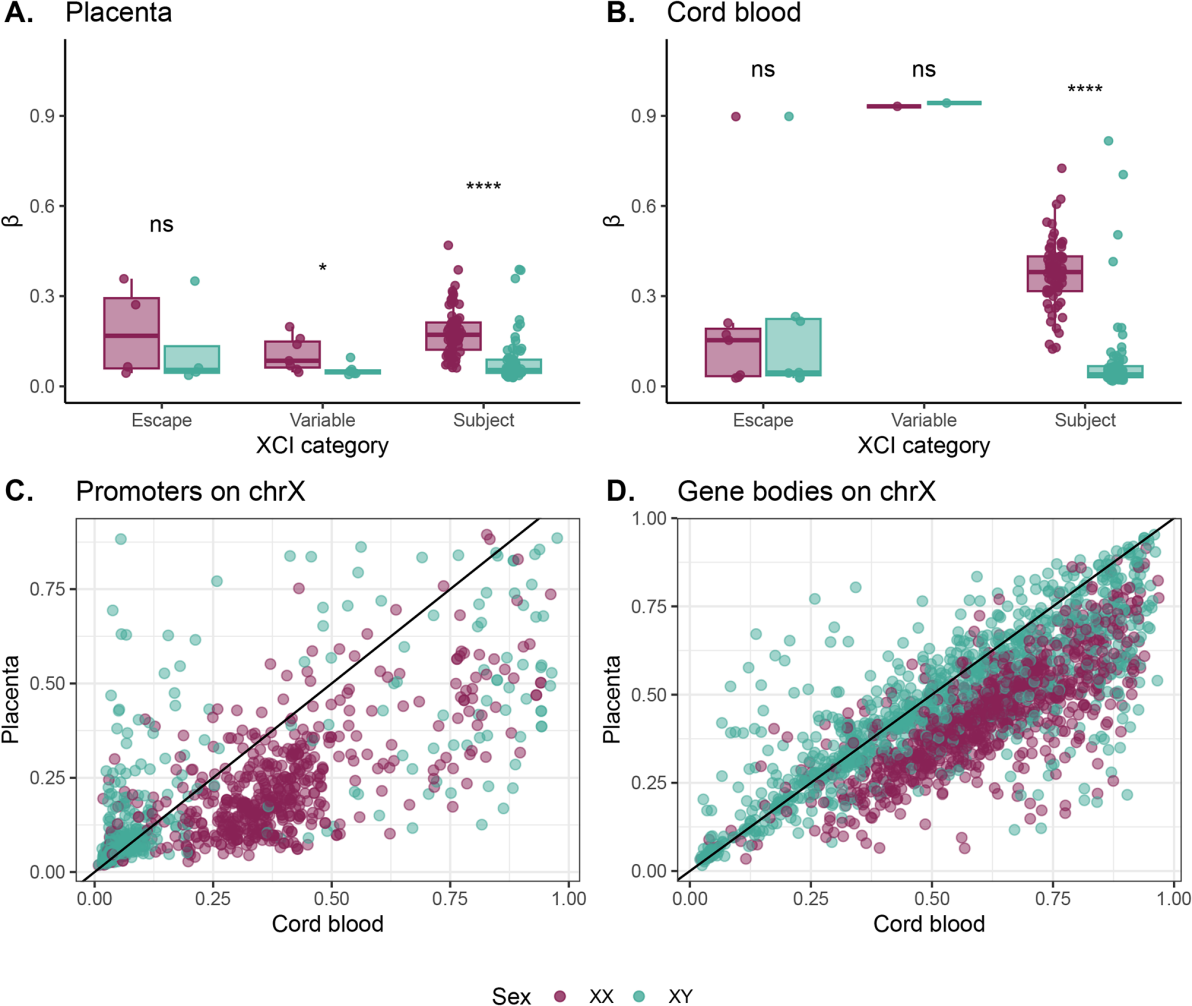
Comparison of XCI-related DNAme between placenta and cord blood. **(A)** Promoter DNAme levels in XX and XY placental samples. Wilcoxon test adjusted p values (FDR) are shown above the plots, ** indicates FDR < 0.01, *** indicates FDR < 0.001, **** indicates FDR < 0.0001, n.s. indicates not significant. **(B)** Promoter DNAme levels in XX and XY cord blood samples. Wilcoxon test adjusted p values (FDR) are shown above the plots, ** indicates FDR < 0.01, *** indicates FDR < 0.001, **** indicates FDR < 0.0001, n.s. indicates not significant. **(C)** Placenta mean β per promoter versus cord blood mean β at the same promoter. Data from both sexes are shown on this plot, with promoter DNAme levels in XX samples shown in purple, and promoter DNAme levels in XY samples shown in teal. **(D)** Cord blood mean β per promoter versus cord blood mean β at the same promoter. Data from both sexes are shown on this plot, with promoter DNAme levels in XX samples shown in purple, and promoter DNAme levels in XY samples shown in teal

At all 78 genes considered, overall promoter DNAme in placenta was low in both sexes across all XCI categories (mean XX β = 0.17, mean XY β = 0.08), though promoters subject to XCI had higher mean DNAme in XX compared to XY placentas (Wilcoxon test FDR < 0.05) indicating accumulation of DNAme on the Xi in XX samples (Fig. 6A). Variable escape promoters also broadly had higher DNAme in XX than XY samples (Wilcoxon test FDR < 0.05).

In contrast, cord blood showed no significant sex difference in DNAme at escape genes, but at promoters subject to XCI, XX samples had higher DNAme than XY samples (Wilcoxon test FDR < 0.05) (Fig. 6B); too few variable escape genes were represented to draw definitive conclusions about this category (*n* = 2). At promoters subject to XCI, the sex difference in DNAme was also larger in cord blood (mean ∆β_XX −XY cord blood_ = 0.30) than in placenta (mean ∆β_XX– XY placenta_ = 0.11), indicating a greater level of DNAme on the Xi in cord blood (Fig. 6B). At escape gene promoters, both tissues showed DNAme levels close to parity between XX and XY samples, indicating minimal DNAme on the Xi.

Comparing DNAme levels between the two tissues, we observed that at X-linked promoters, XX samples exhibited higher DNAme in cord blood than in placenta. In contrast, XY promoter DNAme was quite variable in both tissues (Fig. 6C). Despite the range of promoter DNAme levels observed, in XY samples most promoters (*n* = 61/78, 78%) had low DNAme in both tissues (β_XY_ < 0.10), consistent with their active transcription (from the Xa) (Fig. 6C).

Placenta and cord blood exhibited largely similar gene body DNAme, though in XX samples cord blood was slightly higher methylated than placenta (Fig. 6D). This indicates some accumulation of DNAme on gene bodies on the Xi in cord blood that is absent in placenta, and likely reflects generally higher genomic DNAme levels in cord blood as a whole.

To contextualize our findings, we considered previous work profiling promoter DNAme on the X chromosome in 27 tissues (excluding placenta) [4], which demonstrated that XX promoter DNAme β values > 0.3 were consistently associated with genes subject to XCI across somatic tissues [4]. In placenta, however, we observed that the majority of genes subject to (*n* = 91/104, 88%) or variably escaping XCI (*n* = 18/22, 82%) as determined by ASE had XX promoter DNAme β values < 0.3 in our data. Additionally, Cotton et al. (2015) observed that only escape genes typically had DNAme β values < 0.15 in both sexes [5]. Although our study corroborated low promoter DNAme at escape genes, we also observed that the placenta displays a notable proportion of both variable escape (*n* = 8/22, 36%) and subject genes (*n* = 40/104, 38%) with mean promoter DNAme β < 0.15 in both sexes.

## Discussion

In this study, we evaluated the extent to which common drivers of DNAme variation affect X-linked DNAme in the human placenta. We initially undertook this study as multiple lines of evidence indicated that the human placenta may be an outlier in the landscape of XCI due to its unique and low-methylated Xi DNAme profile. As XCI typically explains a large proportion of DNAme variation on the X chromosome in most tissues [4], we sought to investigate whether this relationship held in placenta, despite lower overall Xi DNAme, and to evaluate the other factors associated with placental X-linked DNAme variation.

Our results confirm earlier work indicating that placental DNAme is not uniformly depleted across the genome, and find that low DNAme is observed at specific regions including PMDs and the Xi [2, 3]. We further demonstrate that the Xi in placenta has less DNAme than other tissues both within and outside of PMDs. Additionally, specific patterns of DNAme on the X chromosome in placenta are distinct as compared to the patterns observed at autosomal loci, though PCA on both autosomal and X-linked DNAme show similar patterns of between-tissue variation, specifically, the separation of XX placentae from other prenatal tissues. We hypothesize that the distinct X chromosome DNAme landscape of the placenta is at least partially attributable to a trophoblast signature, as both placenta and chorion appeared distinct from other tissues– and both derive at least in part from trophectodermal cells [48–50]. Additionally, from the earliest stages of development during DNAme reprogramming, the trophectoderm appears to acquire less *de novo* DNAme than other lineages in a pattern that persists to term, and which we may be observing extended to the X chromosome [51–53]. Coupled with our finding of little variation in X chromosome DNAme across gestation within the placenta, our results thus suggest that X-linked DNAme patterns observed in the placenta are established early in human development, likely after the trophoblast differentiates from other extraembryonic and embryonic tissues.

Within term placentae, we found that cell composition was associated with the first several principal components of X chromosome DNAme in XX samples. Although this parallels what is seen at autosomal DNAme in placenta [24, 25], this is a novel observation for X-linked DNAme. Additionally, we know from previous work that the ratio of cytotrophoblast to syncytiotrophoblast cells may vary by sex in the placenta such that XY placentas have relatively more syncytiotrophoblast and less cytotrophoblast (this cohort [25], as well as [54] and (Beraldo et al., in prep)). Taken together, our results indicate that X chromosome DNAme likely varies between the major placental cell types, and should be considered in future gene expression and DNAme studies. We also observe associations between PC1 and PC2 with maternal age at delivery, placental weight, and birth weight z-score. While the precise meaning of these associations is not yet clear, they indicate lability of X-linked DNAme in the placenta, and provide motivation for studying the sex chromosomes in epigenome-wide association studies going forward.

Our results confirm and extend earlier findings, which indicated a unique DNAme profile of the X chromosome in placenta, particularly in XX samples (Xi). However, a major outstanding question in the literature regarded whether low levels of Xi promoter DNAme in the placenta corresponded with escape from XCI. Allele-specific expression (ASE) data from [10] indicate that at the majority of genes (71%), XCI status in placenta was concordant with XCI status in somatic tissue, and at only 2.7% of genes was XCI status completely discordant (escape/subject or subject/escape) between placenta and somatic tissues. For the remaining 26% of genes investigated, all genes called escape or subject to XCI in either placenta or somatic tissue were called variable escape in the other tissue. Taken together, these results indicate that the placenta does not harbor an excess of genes escaping from XCI. These findings are additionally consistent with a previous cross-tissue XCI study (that did not include placenta), which determined overall consistency in XCI status across tissues, with approximately 5% of genes escaping tissue-specifically [55]. Our results thus provide evidence that even genes subject to or variably escaping XCI display lower levels of promoter DNAme in placenta than would be observed in other tissues, and that promoter DNAme on the Xi may not be critical for the maintenance of XCI at many genes in the placenta, as it is in other tissues. In both the discovery and replication cohorts, for genes reported to be subject to XCI in the majority informative individuals (proportion subject > 0.9), we observed promoter DNAme to be highly variable. While the factors underlying this variation in DNAme are not yet understood, this signature appears to underscore the absence of a strong relationship between DNAme and XCI. Our observation of high DNAme on the Xi in the promoter of XCI-escapee *FLNA* provides further evidence that Xi DNAme does not correlate well with XCI in placenta, as genes can escape with high Xi promoter DNAme, and be subject to or variably escape XCI with very low Xi promoter DNAme.

Our study is limited by a lack of allele-resolved DNAme measurements, which complicates the interpretation of X-linked DNAme signatures. In XX samples, X chromosome DNAme array profiles represent the averaged signal of two distinct chromatin environments (Xa, Xi) [5, 56]. By comparing XX samples to XY samples (which harbor only an Xa), we can resolve some of this signal to infer that the patterns seen only in XX samples must arise from the Xi. However, future research should prioritize techniques such as long-read sequencing to better differentiate the Xi and Xa DNAme profiles, both in placenta and in other tissues. Additionally, incorporating matched DNAme and RNA expression data from the same placentas would strengthen conclusions regarding the relationship between DNAme and XCI in placenta. However, given the variability of XCI within a single placenta, careful experimental design will be critical, as extremely skewed XCI is common within clonally-derived chorionic villous trees, and skewing in opposite directions is frequently seen across the same placenta [10, 48, 57]. Further, DNAme and RNA sequencing data from this heterogeneous tissue are frequently collected by pooling multiple villous sites, which should be avoided for accurate XCI studies [58].

As DNAme is acquired late in the process of XCI [59, 60], it is possible that low Xi DNAme in placenta may indicate reliance on earlier-acquired mechanisms for XCI maintenance; evidence supporting this conclusion comes from imprinted XCI in mouse extraembryonic tissues, for which DNAme is not required [59]. Considering other possible players in the placental XCI landscape, post-translational histone modifications are understudied in the placenta, including on the X chromosome, and should be investigated. MicroRNA species are understudied in relation to XCI in all tissues [61], but have been proposed to act as post-transcriptional modifiers of X-linked gene expression [62], and are important players in placental gene regulation, especially the imprinted microRNA clusters on chromosomes 14 and 19 [63, 64]. Additionally, it was recently reported that *XIST*, the long noncoding RNA responsible for XCI establishment, is a key player in the maintenance of XCI in human B cells, specifically at genes lacking promoter DNAme [65]. As the earliest player in XCI, the localization of *XIST* should perhaps be more closely investigated on the placental Xi.

Overall, our results demonstrate the human placenta is an outlier in terms of its X chromosome DNAme landscape, and indicate that the distinct placental DNAme profile does not correspond with widespread escape from XCI. Future work should seek to investigate other factors associated with XCI in placenta, and separately to understand the dynamics of early development that lead to the distinct Xi DNAme profile in placenta. Other factors to consider related to XCI may include *DNMT1* (mouse knockout leads to X chromosome hypomethylation [66]), *DNMT3B* (critical for LINE-1 DNAme on the Xi [67]), and *SMCHD1* (recruited by *Xist*, knockout mice show decreased X chromosome promoter DNAme [59]), among others.

## Methods

### Cohort construction

To enable investigation into the X chromosome DNA methylation (DNAme) profile of the placenta compared to other tissues and across gestation, we compiled DNAme array data from several datasets, and separated the data into three cohorts: (i) a *multi-tissue* dataset (Table 1), from which placenta (chorionic villus) samples were further subdivided into (ii) a *discovery cohort* profiled with Illumina Infinium MethylationEPIC v1.0 (EPIC) array (Table 2) and (iii) a *replication cohort* profiled with the Illumina Infinium HumanMethylation450 (450K) array (Supplementary Table 1). For cohort details see Methods.

The *multi-tissue* dataset (Table 1) included placental samples from all trimesters of pregnancy, fetal membranes from the third trimester (chorion, amnion), second trimester fetal somatic tissues (brain, kidney, muscle, spinal cord), and term cord blood samples. EPIC data were pulled from matched amnion, chorion, and placental samples (GSE115508) [12], and cord blood (GSE224339) [68]. 450K data were compiled from preterm placentae and matched fetal somatic tissues (GSE69502) [15], term placentae (GSE55196, GSE71678, GSE75248, GSE98224, GSE100197, GSE108567) [26, 37, 38, 49, 69, 70], and additional cord blood samples (GSE101840, GSE151042) [71], as well as sorted cord blood cell types (GSE68456) [72]. The multi-tissue dataset samples were processed and normalized together, full details of sample inclusion criteria and data processing are outlined in Methods.

For the primary analyses of placental DNAme profiles on the X chromosome, we utilized a *discovery cohort* of normative placentas across gestation, profiled with the EPIC array. These samples were collected in Vancouver, Canada (V-NORM and V-SSRI sub-cohorts) and Queensland, Australia (QF2011 sub-cohort), and represent singleton pregnancies without chromosome abnormalities. While placentas delivered preterm necessarily exhibit a subset of complications, the preterm cases included in this cohort were selected to represent a heterogeneous, normative population of samples not dominated by any one pathology, see Methods. Term samples from this cohort have been previously published under GSE232778 [25]. All samples from the discovery cohort were included in the multi-tissue dataset described above, but for all analyses not based on multiple tissues, data from the discovery cohort were processed and normalized together, without any other samples. For XCI analyses comparing DNAme to allele-specific expression (ASE) data from term placentas, only term samples from the discovery cohort were considered. Full cohort demographic details are presented in Table 2.

Finally, we compiled a *replication cohort* for placental XCI analyses, consisting of all term placental samples from the 450K datasets (subsets of which were also included in the multi-tissue dataset). For replication analyses, DNAme data from the replication cohort were processed and normalized together, without any other samples. Demographic details for the term 450K samples are provided in Supplementary Table 1, with a detailed description of inclusion criteria presented in the Methods.

### Discovery cohort (EPIC)

The major analyses in this study are based on a cross-gestation cohort of normative placentae (chorionic villi) from three study cohorts (V-NORM, V-SSRI, and QF2011) sampled in Vancouver, Canada (V-NORM, V-SSRI) and Queensland, Australia (QF2011), and profiled with the Illumina Infinium MethylationEPIC (“EPIC”) DNAme array at one centre in Vancouver, Canada (randomized during array processing for key technical and biological variables, see [25]. Term samples from this cohort have been previously published under GSE232778. V-SSRI and QF2011 represented term (or near term) deliveries, while V-NORM samples profiled placentas from all trimesters of pregnancy, and term deliveries. Regarding exclusion criteria, V-SSRI excluded mothers with bipolar illnesses, hypertension, current substance abuse, any diabetes, or infants with congenital brain malformations or fetal growth disorders. Term V-NORM excluded any preeclampsia, while preterm V-NORM excluded early onset preeclampsia, HELLP syndrome, and placental mesenchymal dysplasia. QF2011 was not subject to any additional exclusions. Prior to data processing, this cohort comprised of 239 placental samples. Final inclusion samples (*n* = 228, 47% XX) after data processing are described in Table 2.

### Replication cohort (450K)

A cohort consisting of Illumina Infinium HumanMethylation450 (“450K”) DNAme data from > 900 placenta samples was constructed by combining the following datasets: GSE71678 (New Hampshire Birth Cohort), GSE75248 (Rhode Island Child Health Study), GSE74738, GSE75196, GSE98224, GSE100197, GSE108567, and GSE128827 (all Epigenetics in Pregnancy Complications (EPIC - Vancouver) Cohort, Vancouver, BC). All samples had available IDATs, and were read into R version 4.2.2. If the same IDAT file was present in multiple datasets, duplicate copies were excluded (duplicate Sentrix ID/Row combination, *n* = 95). Additionally excluded were any samples born preterm (< 37 weeks of gestational age) (*n* = 127) missing gestational age information (*n* = 1), affected by known or suspected chromosomal abnormalities (*n* = 6), diagnosed with preeclampsia (*n* = 20), or affected by IUGR (*n* = 1). After applying exclusions, 838 samples remained for data processing. Final inclusion samples (*n* = 635, 49% XX) after data processing are described in Supplementary Table 1.

### Multi-tissue cohort

DNAme profiles from a variety of tissues were combined into a multi-tissue dataset. Gestational tissues (placenta, amnion, and chorion) were obtained from GSE115508 (EPIC), fetal somatic tissues (brain, spinal cord, kidney, muscle) and matched placental samples were obtained from GSE69502 (450K). Term umbilical cord blood were obtained from GSE151042 (450K), and additional term umbilical cord samples were obtained from GSE224339 (EPIC). The EPIC discovery cohort described above was combined with this data, as were a subset of the replication cohort’s 450K term placentas.

To have relatively balanced sample numbers per dataset in the multi-tissue cohort, larger datasets were randomly sampled for inclusion in the final cohort. The term 450K placentae were sampled for inclusion in this multi-tissue dataset such that each major contributing cohort (RICHS, NICHD, EPIC– Vancouver) was randomly sampled for 12 samples of each sex. GSE151042 was randomly sampled such that 36 term cord blood samples of each sex were contributed to the final dataset. Finally, from GSE224339 we selected 22 genetically-distinct samples of term cord blood. Samples from all cohorts were subjected to the data processing steps described below, and final inclusion sample numbers by sex are indexed in Table 1.

### DNAme data processing

IDAT files were loaded into R v4.3.1 [73] and data were managed using minfi version 1.46.0, and ewastools version 1.7.2 [74, 75]. First, sample genetic identity was confirmed to be unique in all cases (except for matched samples from GSE69502 and GSE115508) using the ewastools function call_genotypes(). Samples with a value > -4 using the snp_outliers() function were excluded from all datasets; this threshold indicates high probability of sample contamination or poor quality [75]. Sample sex was assessed using X and Y chromosome probe fluorescence intensities with a version of the ewastools function check_sex() modified to work with minfi objects, as described in [56]. One sample with reported sex not matching the DNAme-derived sex was excluded: a chorionic villus sample with a putative mosaic 45,X/46,XX karyotype from GSE71678.

For placental samples, cell composition was assessed using the PlaNET R package, which estimates the proportion of six major placental cell types in bulk chorionic villous DNAme samples (cytotrophoblast, syncytiotrophoblast, stromal, endothelial, Hofbauer, and nucleated red blood cells) [24]. PlaNET estimates first trimester and third trimester/term cell compositions directly, while second trimester cell compositions were estimated by interpolation of the first and third trimester estimates as recommended by the package author (Yuan V, personal communication).

Probe filtering was conducted in a sex-chromosome informed manner, according to our previously published protocol [56]. The same probe filtering criteria were applied to all three cohorts (cross-gestation, multi-tissue, and 450K placentae) separately. Briefly, poor quality probes were defined as those with a detection P value > 0.01, bead count < 3, or missing values, in > 5% of samples. Additionally, probes overlapping polymorphic loci or targeting non-specific sequences were excluded using the “MASK_general” column in the 450K and EPIC annotations from Zhou et al. (2017) [76]. Probes were additionally excluded if they failed to map to hg38 loci [76]. The data were then normalized using adjustedFunnorm() from the wateRmelon package [77] separately in the three cohorts (discovery, replication, and multi-tissue).

Following data processing and normalization, DNAme data were available for 762,551 CpGs (X chromosome CpGs = 16,642) in the discovery cohort, 384,419 CpGs (X chromosome CpGs = 8,676) in the multi-tissue cohort, and 423,517 CpGs (X chromosome CpGs = 9,823) in the replication cohort.

All principal components analyses were run on centred and scaled β value data, with post-hoc testing for associations with linear models run for principal component scores regressed on sample demographic characteristics, summarizing with r^2^ and p values of models. Linear modelling was conducted on X chromosome DNAme β values for gestational age assessments using the R package limma, adjusting for genetic ancestry from the PlaNET package [69, 78]. Effect sizes for the gestational age linear model were set at|∆β| ≥ 0.03, based on technical replicates from this dataset as described in [25].

PC-PR2 analysis was conducted using the pcpr2 R package [79] on centered and scaled X chromosome DNAme β values in XX samples from the multi-tissue dataset, with the proportion of variability explained set to 0.9. Covariates analyzed included collapsed tissue and dataset variables, to reduce collinearity between tissue and GEO dataset. In brief, tissues were grouped into (i) placenta, (ii) fetal membranes (amnion, chorion), (iii) fetal somatic tissues (brain, kidney, muscle, spinal cord), and (iv) blood (cord blood, sorted cord blood). Dataset was collapsed based on the GEO dataset owner as all datasets originated from one of four laboratories (Fradin, Marsit, Plusquin, Robinson).

### Functional annotation of CpGs

A list of all candidate promoter-like elements (PLS) in hg38 was downloaded from the University of California, Santa Cruz (UCSC) Genome Browser (encodeCcreCombined table) in August 2022 [80]. To identify corresponding genes, the genomic coordinates of the PLS list were overlapped with the coordinates all transcription start site (TSS) hg38 coordinates in GENCODE v41 (downloaded August 2022 [81]), using the mergeByOverlaps() function from the GenomicRanges package [82]. ENCODE promoters were assigned to genes if the promoter was ± 200 base pairs of a GENCODE TSS, based on the ENCODE definition of PLS. This yielded 24,448 promoter-gene associations, genome-wide. The promoter coordinates were further overlapped with hg38 coordinates of CpG islands (CGI), downloaded from the UCSC Genome Browser (cpgIslandExtUnmasked table); CGI were classified into high-density (HC) and intermediate-density (IC) islands using the Weber et al. (2007) criteria (HC island: > 500 base pairs CG content > 55%, and observed/expected CpG ratio > 0.75; IC island: > 200 base pairs, CG content > 50%, observed/expected CpG ratio > 0.48) [5, 83].

Promoters were further overlapped with the coordinates of CpGs interrogated by the probes on the EPIC array. Using mergeByOverlaps(), we identified 72,917 CpGs in 18,101 promoters genome-wide, and 2,251 CpGs in 591 X chromosome promoters.

Gene body hg38 coordinates from GENCODE v41 (for protein-coding and long noncoding RNA genes) were similarly overlapped with the coordinates of probes on the EPIC array. This resulted in 636,470 CpGs in 33,369 genes (protein-coding and long noncoding RNA) genome-wide. On the X chromosome this overlap yielded 12,383 CpGs in 986 unique genes.

A list of proximal enhancer-like elements (pELS) in hg38 was extracted from the same table as the PLS elements (encodeCcreCombined). Based on the ENCODE definition of pELS, we annotated enhancers to genes with transcription start sites within 2 kilobase windows of the pELS, yielding 123,528 enhancer-gene associations in hg38. We further overlapped these enhancers with the coordinates of CpGs included on the EPIC array yielding 75,778 CpGs in 46,277 enhancers genome-wide, and 1,568 CpGs in 1,002 X chromosome enhancers.

All genomic CpGs were also indexed for overlap with partially-methylated domains using the coordinates provided in [2] lifted over from hg18 to hg38. These coordinates were overlapped with the EPIC array, resulting in 76,035 CpGs genome-wide in PMDs, including 2,288 X chromosome CpGs. EPIC array CpG coordinates were additionally overlapped with the coordinates of repetitive elements using the hg38 RepeatMasker (rmsk) track from the UCSC Genome Browser, downloaded January 2023 [84, 85]. CpGs in repetitive elements from the LINE-1 and SINE families were identified using the repClass and repFamily columns of the RepeatMasker annotation, and included for LINE-1 elements 10,584 CpGs genome-wide, and 270 X chromosome CpGs. SINE elements overlapped 15,026 CpGs genome-wide, and 291 CpGs on the X chromosome.

### Annotation of DNAme to allele-specific expression XCI data

For XCI analyses, promoters and gene bodies that we had identified as overlapping CpGs in our final DNAme datasets were overlapped with the list of genes with XCI status in placenta (chorionic villi), reported in [10]. The mean and median DNAme β value of all CpGs within a promoter or gene body were calculated per-sex, per-promoter/gene body, and tested for correlation with the ASE XCI metrics proportion subject or median allele balance using Pearson correlation tests.

### Statistical tests

All appropriate statistical tests were adjusted for multiple comparisons using the Benjamini-Hochberg false discovery rate (FDR) adjustment method.

## Electronic supplementary material

Below is the link to the electronic supplementary material.

Supplementary Material 1

## Data Availability

The DNA methylation datasets supporting the conclusions of this manuscript are all publicly available, as described in Methods. Additional DNA methylation samples published for the first time in this manuscript have been made available under GSE281173. Analysis code generated during this study is available at https://github.com/amy-inkster/Placenta-DNAme-XCI.
