# Supplementary material for: Breaking rules: the complex relationship between DNA methylation and X-chromosome inactivation in the human placenta
Source: Biol Sex Differ. 2025 Mar 4;16:18. doi: 10.1186/s13293-025-00696-6 (PMC11877730; doi:10.1186/s13293-025-00696-6)

Supplementary Information for:

Breaking Rules: the complex relationship between DNA methylation and X-chromosome inactivation in the human placenta

Amy M Inkster[1,2], Allison M Matthews[1,3], Tanya N Phung[4], Seema B Plaisier[4], Melissa A Wilson[4], Carolyn J Brown[2], Wendy P Robinson[1,2]

1. BC Children's Hospital Research Institute, 950 W 28th Ave, Vancouver BC, V6H 3N1, Canada.
2. Department of Medical Genetics, University of British Columbia, 4500 Oak St, Vancouver BC, V6H 3N1, Canada.
3. Department of Pathology & Laboratory Medicine, University of British Columbia, 221 Wesbrook Mall, Vancouver BC, V6T 1Z7, Canada.
4. Center for Evolution and Medicine, School of Life Sciences, 427 E Tyler Mall, Tempe AZ, 85281, USA.

Supplementary Table 1. Demographic characteristics of 450K replication cohort samples, profiled with the Illumina Infinium HumanMethylation450 array.

	XX (female)	XY (male)	p value*
**n**	309	326	
**Cohort**			
Epigenetics in Pregnancy Cohort (GSE55196, GSE98224, GSE100197, GSE108567)	25 (8.1)	29 (8.9)	ns
New Hampshire Birth Cohort (GSE71678)	144 (46.6)	159 (48.8)	
Rhode Island Child Health Study (GSE75248)	140 (45.3)	138 (42.3)	
**Gestational age at delivery (mean (SD))**	39.4 (1.1)	39.5 (1.1)	ns
**PlaNET Ancestry****			
Coordinate 1 (mean (SD))	0.05 (0.2)	0.02 (0.1)	ns
Coordinate 2 (mean (SD))	0.05 (0.2)	0.02 (0.1)	ns
Coordinate 3 (mean (SD))	0.91 (0.3)	9.95 (0.2)	<0.05
**Estimated cell composition**††			
nRBC (mean (SD))	0.03 (0.02)	0.03 (0.02)	ns
Hofbauer (mean (SD))	0.01 (0.01)	0.01 (0.02)	ns
Endothelial (mean (SD))	0.06 (0.04)	0.07 (0.04)	ns
Stromal (mean (SD))	0.06 (0.05)	0.06 (0.05)	ns
Cytotrophoblast (mean (SD))	0.09 (0.08)	0.09 (0.07)	ns
Syncytiotrophoblast (mean (SD))	0.74 (0.17)	0.74 (0.16)	ns

*p values represent significance of ANOVAs for continuous variables and Chi square tests for categorical variables.
†Birthweight Z-score refers to the number of standard deviations away from the sex- and gestational age-specific Canadian population mean presented in Kramer et al. (2001) (22).
**Genetic ancestry was estimated using the PlaNET R package, and is described on three compositional axes, Coordinate 1 reflects probability of African ancestry, Coordinate 2 reflects probability of East Asian Ancestry, and Coordinate 3 reflects probability of European ancestry.
††Cell composition of bulk chorionic villus tissue estimated using the PlaNET R package.

Supplementary Table 2. Summary of Pearson correlation tests between DNAme metrics at gene promoters and XCI allele-specific expression (ASE) metric "proportion subject" (proportion of informative individuals in which a gene was measured to be subject to XCI), or median allele balance, both ASE metrics from Phung et al. 2022. For all genes on the X chromosome covered in both Phung et al. (2022) and the term DNAme discovery cohort, correlation was tested between summarized DNAme per gene and proportion subject or median allele balance. Δβ refers to the sex difference in mean DNAme, and is calculated as Δβ = mean XX β – mean XY β. HC and IC refer to high- and intermediate-density CpG island classes. TSS indicates transcription start site and reflects base pair windows upstream of the TSS. Pearson correlation test statistics (correlation coefficient, p value, and adjusted p values (FDR) are shown for all tests.

**Proportion Subject**			
Promoters	R	p value	FDR
Median XX β	0.16	0.07	0.08
Mean XX β	0.18	0.04	0.06
Δβ	0.27	0.003	0.004
Δβ (excluding promoters where mean XY β > 0.10)	0.29	0.003	0.004
Δβ (HC or IC & excluding promoters where mean XY β > 0.10)	0.33	0.001	0.004
Gene bodies	R	p value	FDR
Δβ	-0.046	0.6	0.6
TSS windows	R	p value	FDR
TSS200 Δβ	0.14	0.06	0.07
TSS200 – TSS1500 Δβ	0.25	0.002	0.004
Enhancers	R	p value	FDR
Δβ	0.25	0.003	0.004
**Median Allele Balance**			
Promoters	R	p value	FDR
Median XX β	0.16	0.02	0.02
Mean XX β	0.18	0.007	0.01
Δβ	0.21	0.001	0.003
Δβ (excluding promoters where mean XY β > 0.10)	0.25	0.0006	0.003
Δβ (HC or IC & excluding promoters where mean XY β > 0.10)	0.26	0.001	0.003
Gene bodies	R	p value	FDR
Δβ	-0.04	0.5	0.5
TSS windows	R	p value	FDR
TSS200 Δβ	0.15	0.01	0.01
TSS200 – TSS1500 Δβ	0.20	0.002	0.004
Enhancers	R	p value	FDR
Δβ	0.18	0.007	0.01

Supplementary Table 3. Results of transcription factor motif enrichment analysis with CentriMo. Columns indicate the transcription factor and chromosome from which it is encoded, motif ID and consensus seq indicate the name and consensus sequence of the enriched motif. E value refers to the central enrichment test statistic in the background set of CpGs, adj p value refers to the statistical significance of the enriched motif in the background set of CpGs, adjusted for multiple tests. Fisher adj p value refers to the differential enrichment test statistic, of the test set (low methylated CpGs on the X in XX placentas) relative to the background set of all processed X chromosome CpGs. A non-significant Fisher adj p value indicates no significantly different enrichment in the test set relative to the background, and is indicated by “n.s.” (Fisher adj p > 0.05).

Transcription Factor	chr	Motif ID	Consensus Seq	E-value	Adj p value	Fisher adj p value
MECP2	X	MECP2_HUMAN.H11MO.0.C	SCCGGRR	1.80E-31	4.50E-34	n.s.
ZBTB33	X	KAISO_HUMAN.H11MO.0.A	SARRYCTCGCGAGAV	3.70E-14	9.10E-17	n.s.
AHR	7	AHR_HUMAN.H11MO.0.B	DTYGCGTGM	1.90E-12	4.70E-15	n.s.
ARNT	1	ARNT_HUMAN.H11MO.0.B	STACGTGMC	1.10E-11	2.80E-14	n.s.
E2F4	16	E2F4_HUMAN.H11MO.0.A	SRGGGCGGGAARD	2.70E-11	6.60E-14	n.s.
MBD2	18	MBD2_HUMAN.H11MO.0.B	SSGKCCGGMGR	2.50E-06	6.30E-09	n.s.
E2F1	20	E2F1_HUMAN.H11MO.0.A	RRNKGGCGGGAARR	3.00E-06	7.50E-09	n.s.
TFDP1	13	TFDP1_HUMAN.H11MO.0.C	RRRRGGCGGGAARN	1.20E-04	3.10E-07	n.s.
E2F2	1	E2F2_HUMAN.H11MO.0.B	GGCGCGAAAC	1.40E-04	3.40E-07	n.s.
SP2	17	SP2_HUMAN.H11MO.0.A	GGSSVGGGGGCGGGGCCDGSGS	3.70E-04	9.10E-07	n.s.
EPAS1	2	EPAS1_HUMAN.H11MO.0.B	VTACGTGMC	7.50E-04	1.90E-06	n.s.
HIF1A	14	HIF1A_HUMAN.H11MO.0.C	GDACGTGM	1.20E-03	3.10E-06	n.s.
BHE40	3	BHE40_HUMAN.H11MO.0.A	DGCACGTGAS	1.50E-03	3.80E-06	n.s.
MYCN	2	MYCN_HUMAN.H11MO.0.A	RGCCACGTGSDS	1.90E-03	4.80E-06	n.s.
ATF3	1	ATF3_HUMAN.H11MO.0.A	GGTSACGTGAB	2.80E-03	7.00E-06	n.s.
KLF12	13	KLF12_HUMAN.H11MO.0.C	VGGGGCGGGGC	2.50E-02	6.20E-05	n.s.
KLF4	9	KLF4_HUMAN.H11MO.0.A	WGGGYGKGGC	3.50E-02	8.70E-05	n.s.
SP3	2	SP3_HUMAN.H11MO.0.B	SGVVGGGGGCGGGGCBRGSS	3.60E-02	9.00E-05	n.s.

Supplementary Figure 1. Placenta versus somatic tissues by dataset and on the autosomes. (A) Principal components analysis of X chromosome DNAme in XX samples, colored by simplified Dataset and Tissue groups, to illustrate the covariates assessed for contribution to DNAme variance by PC-PR2 analysis. Points are colored by simplified dataset (Fradin, Marsit, Plusquin, Robinson) and point shapes represent simplified tissue (placenta, fetal membranes, fetal somatic, and cord blood). **(B)** Density plot of autosomal DNAme in XX samples, with density lines colored by tissue. The density plot of placenta is additionally shaded in purple. **(C)** Density plot of autosomal DNAme in XY samples, with density lines colored by tissue. The density plot of placenta is additionally shaded in purple. **(D)** Principal components analysis of autosomal DNAme in XX + XY combined samples, colored by tissue. **(E)** Principal components analysis of X chromosome DNAme in XX + XY combined samples, colored by tissue.

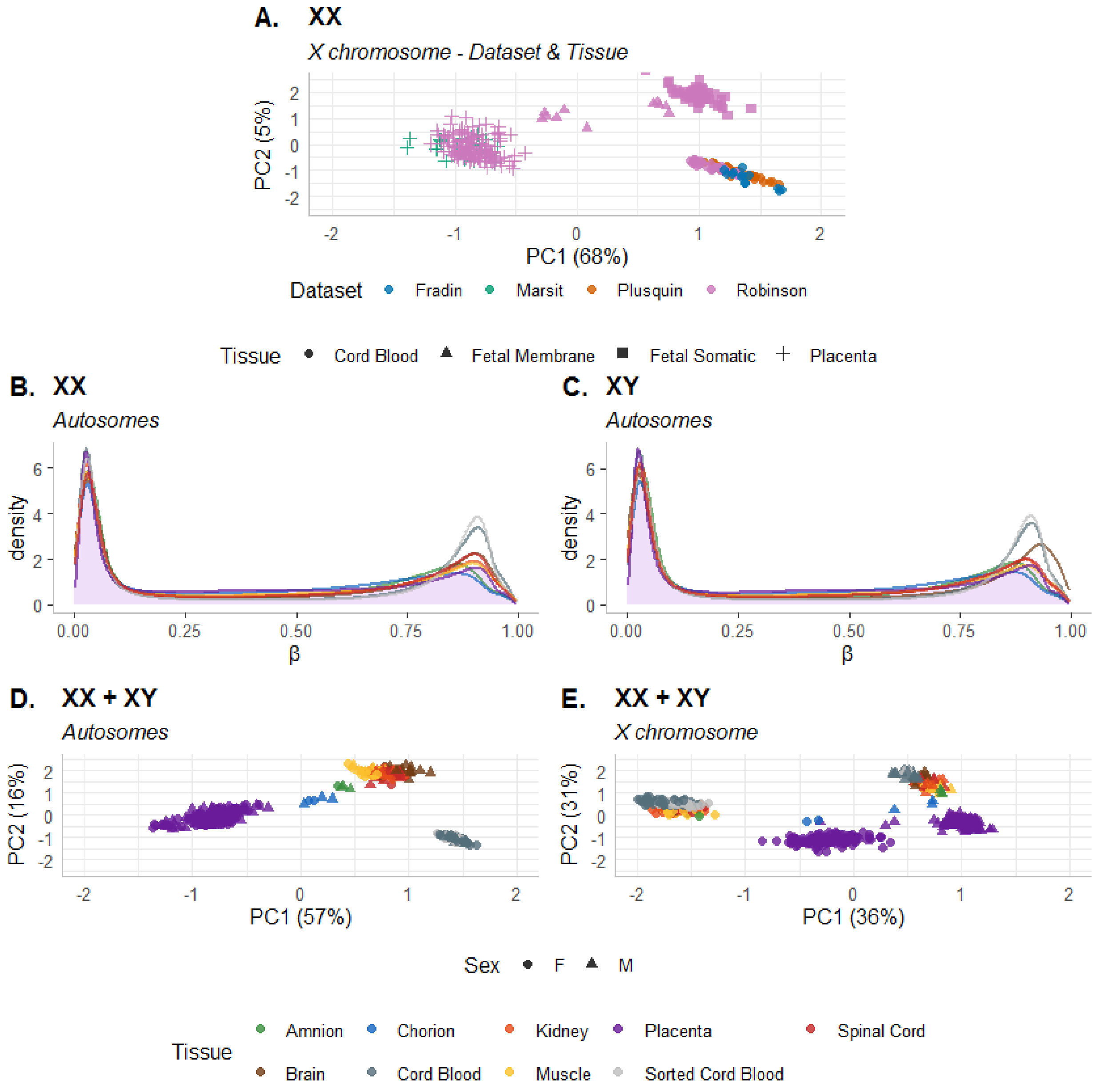

Supplementary Figure 2. LINE-1 and SINE elements have lower DNAme in placenta than somatic tissues, explained by overlap with PMD elements except on the X in XX samples. For simplicity, “somatic” is depicted as the mean of amnion, muscle, and cord blood samples, and is shown in grey. Placental DNAme levels are shown in purple. PMD indicates partially methylated domains. Wilcoxon test statistics indicate whether mean DNAme differs between placenta and somatic tissue. **** indicates $p < 0.0001$, *** $p < 0.001$, ** $p < 0.01$, * $p < 0.05$, n.s. indicates $p > 0.05$. **(A)** Autosomal LINE-1 DNAme values in PMD and non-PMD regions. **(B)** X chromosome LINE-1 DNAme values in PMD and non-PMD regions. **(C)** Autosomal SINE DNAme values in PMD and non-PMD regions. **(D)** X chromosome LINE-1 DNAme values in PMD and non-PMD regions.

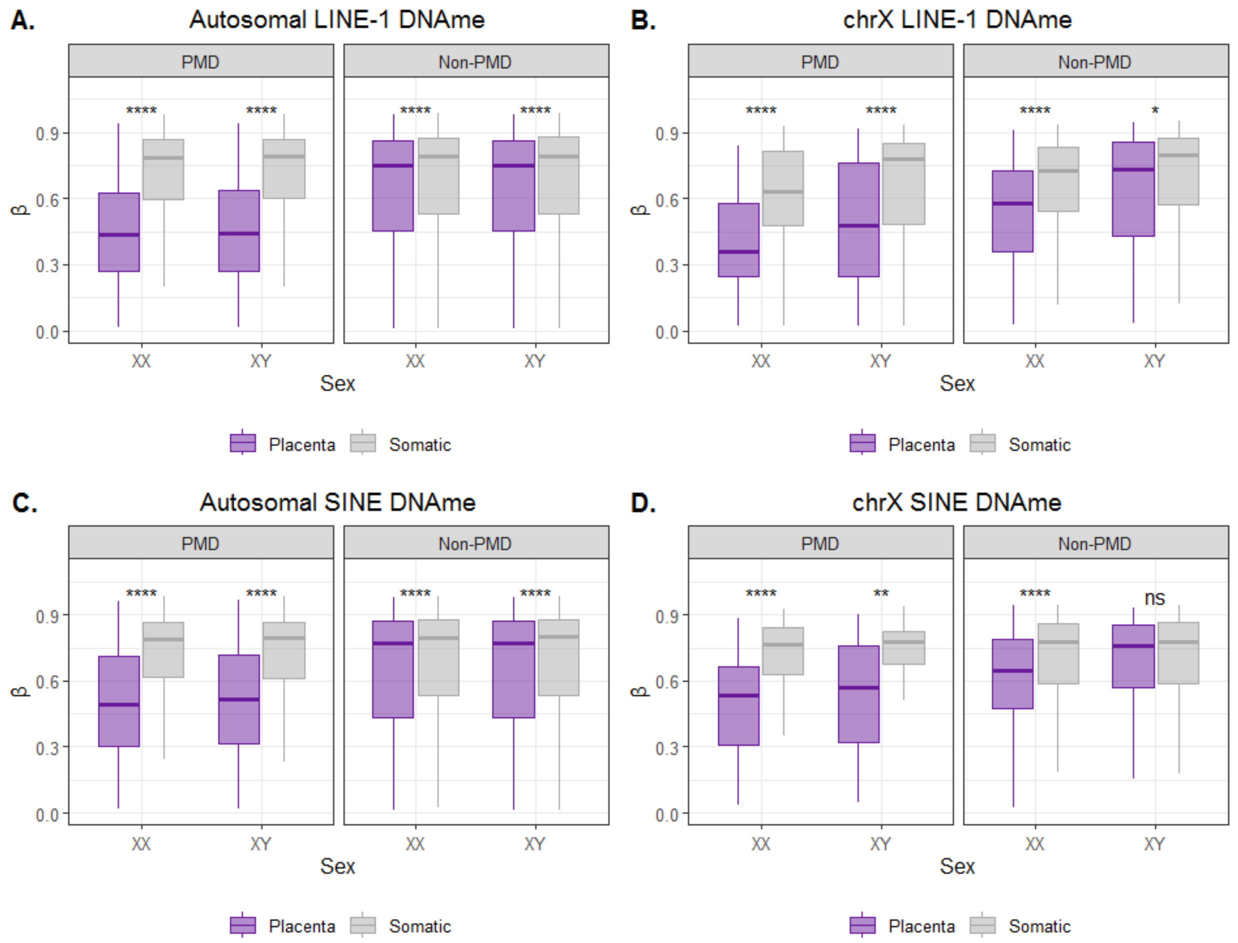

Supplementary Figure 3. DNAme on the X chromosome in XX samples is depleted outside of both PMDs and repetitive elements. Bottom right panel is key where X in females is lower in placenta, but not in males, implying that placenta Xi is depleted and not Xa). For simplicity, “somatic” is depicted as the mean of amnion, muscle, and cord blood samples, and is shown in grey. Placental DNAme levels are shown in purple. PMD indicates partially methylated domains. Wilcoxon test statistics indicate whether mean DNAme differs between placenta and somatic tissue. **** indicates p < 0.0001, *** p < 0.001, ** p < 0.01, * p < 0.05, n.s. indicates p > 0.05. **(A)** X chromosome DNAme in repetitive elements (LINE-1 or SINE) and overlapping PMD or non-PMD regions, **(B)** Autosomal DNAme in repetitive elements (LINE-1 or SINE) and overlapping PMD or non-PMD regions.

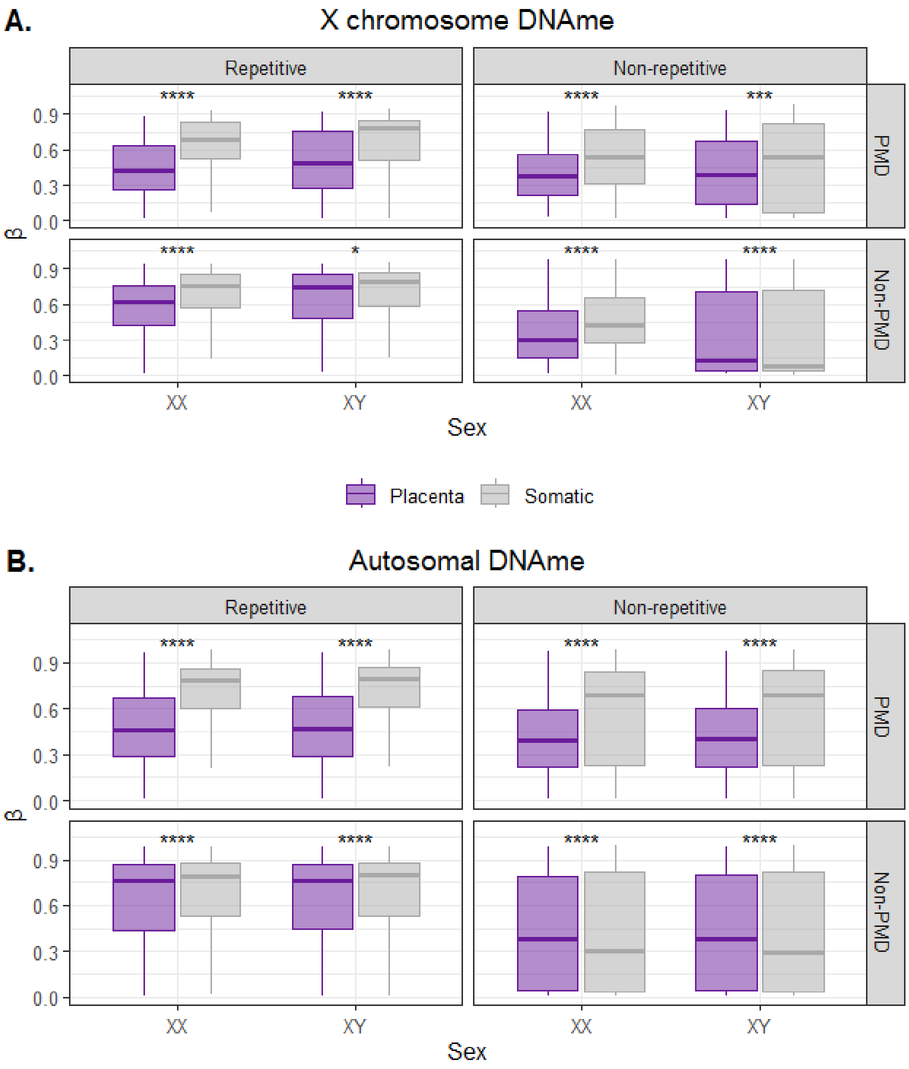

Supplementary Figure 4. Principal components analysis and cell composition in each sub-cohort of the EPIC discovery cohort. (A) Heatmap of the strength of association (PC ~ variable) in XX samples from the QF2011 sub-cohort, shading reflects r^2 while black dots represent significant associations at a nominal $p < 0.05$, with larger dots indicating more significance. Epi age accel refers to epigenetic age acceleration, Plac refers to placenta, BW SD refers to the z-score of birth weight for sex and gestational age, Prob African/Asian/European refers to the three continuous ancestry components estimated by PlaNET, and Cyto/Synctio reflects the ratio of estimated cytotrophoblast over syncytiotrophoblast cells, per sample. **(B)** Heatmap of the strength of association (PC ~ variable) in XX samples from the V-NORM sub-cohort. Figure elements are plotted to be consistent with those described in panel A. **(C)** Heatmap of the strength of association (PC ~ variable) in XX samples from the V-SSRI sub-cohort. Figure elements are plotted to be consistent with those described in panel A. **(D)** Comparison of cytotrophoblast/syncytiotrophoblast ratio in XX samples between the three sub-cohorts. Wilcoxon test nominal p values are indicated above horizontal lines for significant comparisons, non-significant comparisons are not shown.

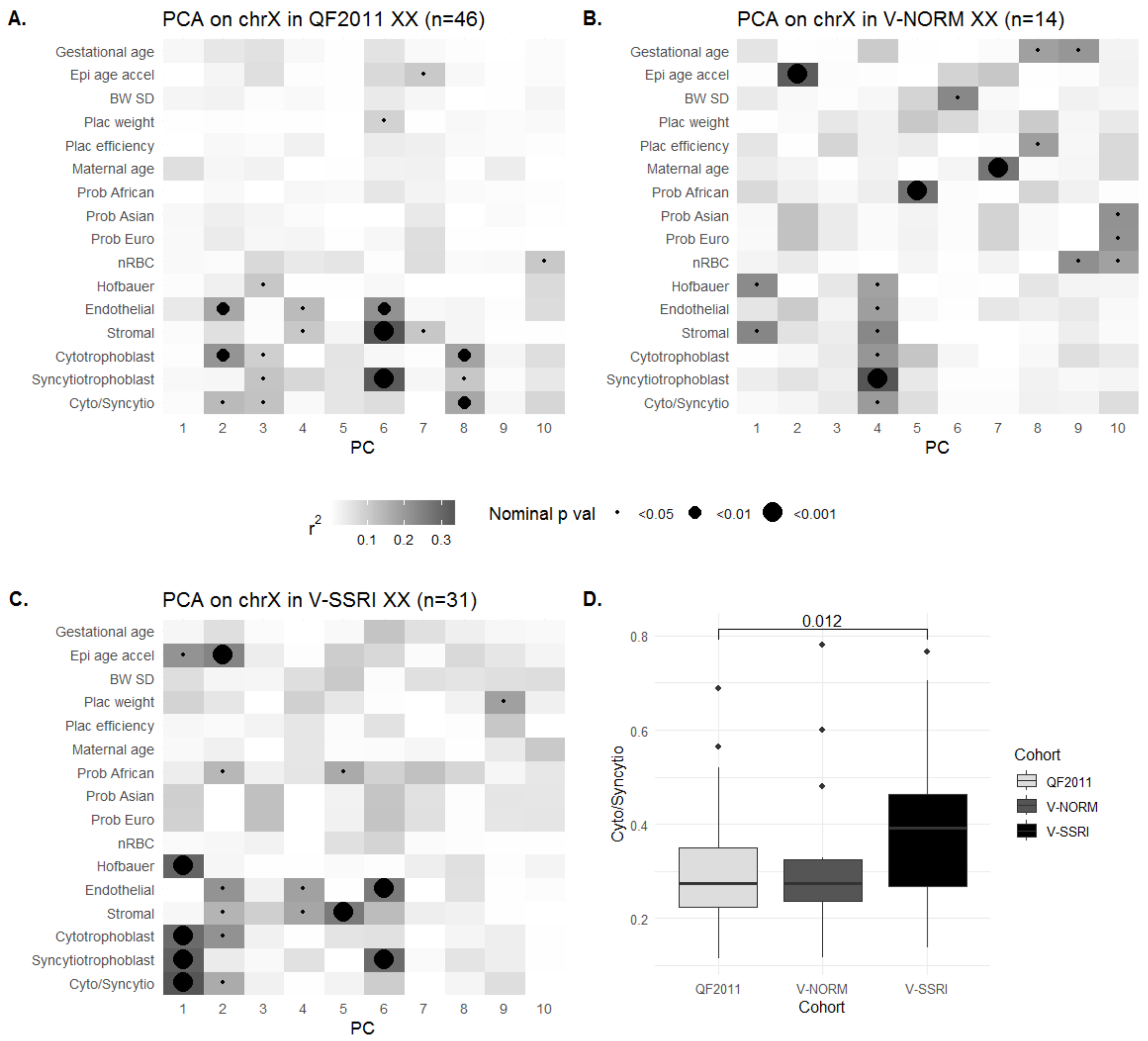

Supplementary Figure 5. Replication of the relationship between DNAme and XCI status from allele-specific expression (ASE) data. (A) Correlation between sex difference in DNAme at X-linked promoters in the 450K replication cohort and proportion subject, considering only promoters that overlap CpG islands and have XY DNAme β < 0.10. Pearson correlation coefficient and p value are shown in the top left corner, a line of best fit is shown in blue with 95% confidence intervals shaded in grey. **(B)** Mean sex difference in DNAme in the 450K replication cohort, at X-linked promoters of genes that are categorized as escape, variable escape, or subject to XCI in (10). Wilcoxon test adjusted p values (FDR) are shown at top of plots, ** indicates FDR < 0.01, *** indicates FDR < 0.001, **** indicates FDR < 0.0001, n.s. indicates not significant.

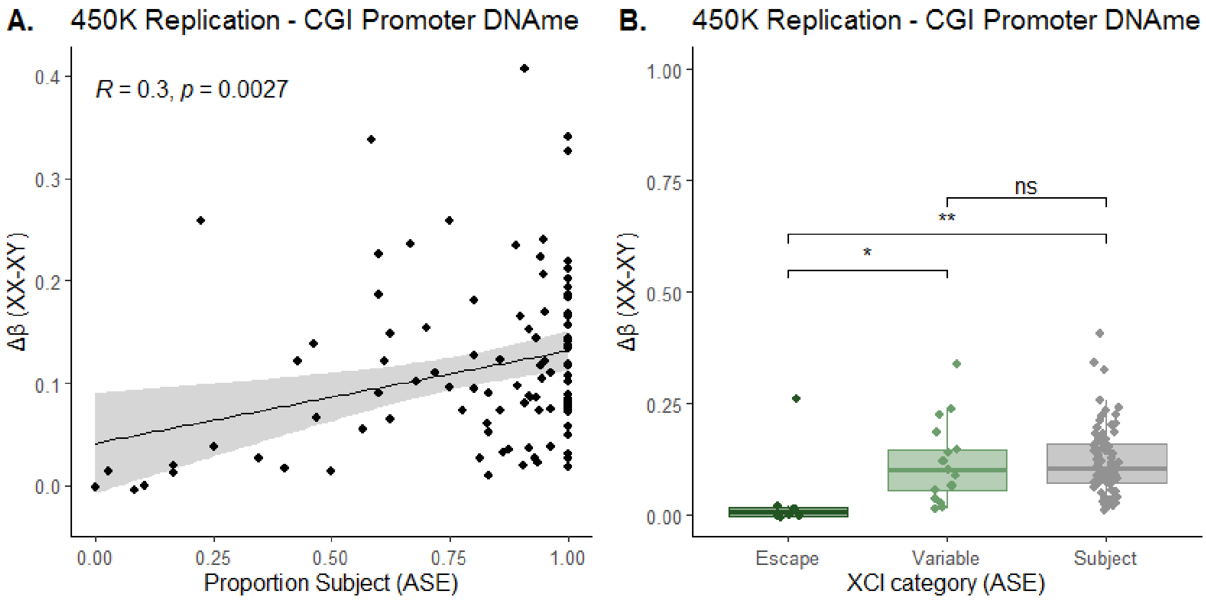

Supplementary Figure 6. Relationship between DNAme in the three sub-cohorts of the discovery cohort and XCI status from allele-specific expression (ASE) data. (A) Correlation between sex difference in DNAme at X-linked promoters in the three sub-cohorts (V-NORM, V-SSRI, and QF-2011) and proportion subject, considering only promoters that overlap CpG islands and have XY DNAme $\beta < 0.10$. Pearson correlation coefficient and p value are shown in the top left corner, a line of best fit is shown in blue with 95% confidence intervals shaded in grey. **(B)** PlaNET cell composition estimates across the three sub-cohorts, each vertical bar represents a single sample, color fill represents the estimated proportion of each estimated cell type, nRBC indicates nucleated red blood cells.

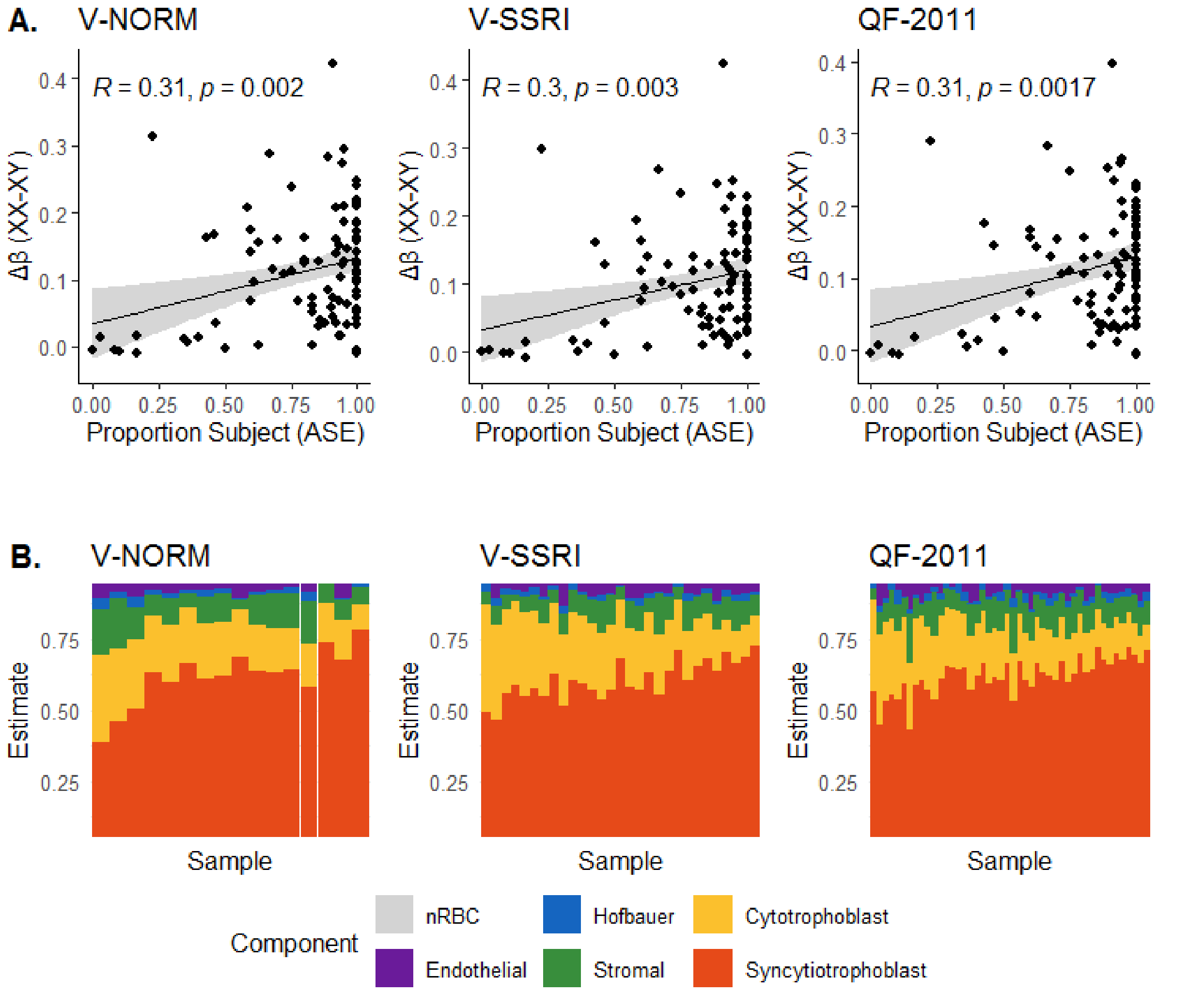

Supplementary Figure 7. Relationship between DNAme in the discovery cohort and gene expression from the X chromosome (not allele-resolved). For all plots gene expression is shown as log2CPM, where counts per million (CPM) have been log2 transformed with an offset of 0.25 + library size. **(A)** Correlation between DNAme at X-linked promoters in XX samples and log2CPM expression of the corresponding genes, Pearson correlation coefficient and p value are shown in the top left corner, a line of best fit is shown in blue with 95% confidence intervals shaded in grey. **(B)** Correlation between DNAme at X-linked promoters in XY samples and log2CPM of the corresponding genes, Pearson correlation coefficient and p value are shown in the top left corner, a line of best fit is shown in blue with 95% confidence intervals shaded in grey.

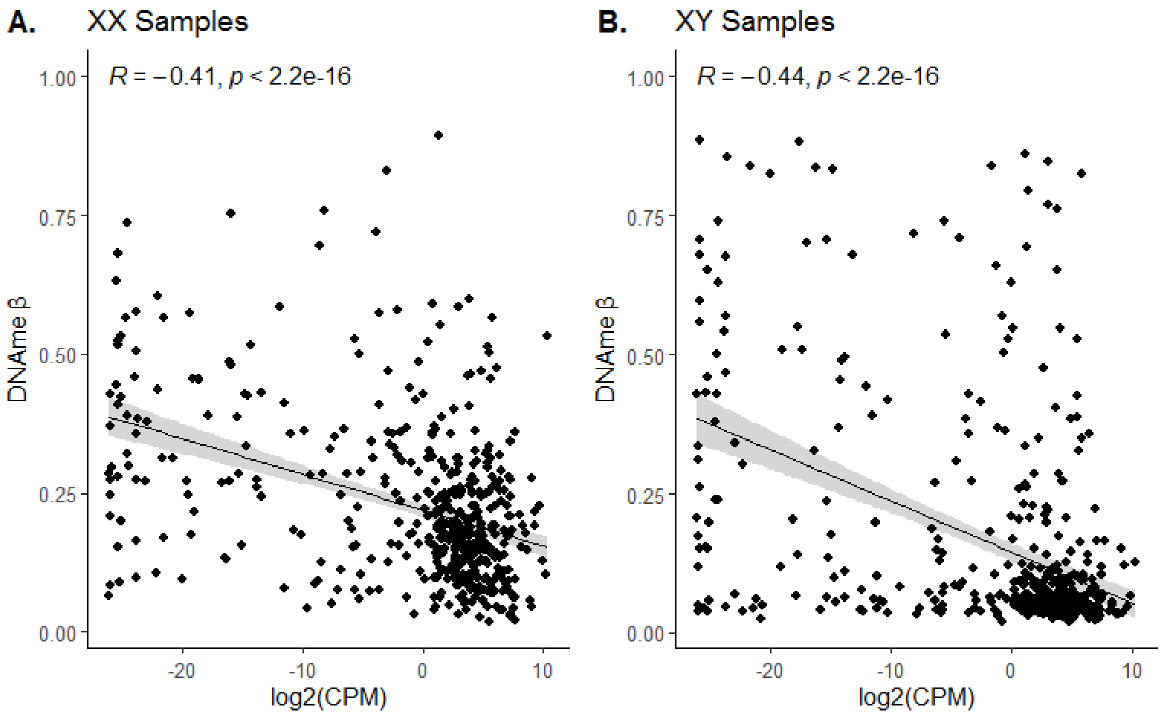

Supplement: Supplementary file 1 — Supplementary Material 1 [file 13293_2025_696_MOESM1_ESM.pdf]
